# The Extra-Virgin Olive Oil Polyphenols Oleocanthal and Oleacein Counteract Inflammation-Related Gene and miRNA Expression in Adipocytes by Attenuating NF-κB Activation

**DOI:** 10.3390/nu11122855

**Published:** 2019-11-21

**Authors:** Sara Carpi, Egeria Scoditti, Marika Massaro, Beatrice Polini, Clementina Manera, Maria Digiacomo, Jasmine Esposito Salsano, Giulio Poli, Tiziano Tuccinardi, Stefano Doccini, Filippo Maria Santorelli, Maria Annunziata Carluccio, Marco Macchia, Martin Wabitsch, Raffaele De Caterina, Paola Nieri

**Affiliations:** 1 Department of Pharmacy, University of Pisa, 56126 Pisa, Italy; beatrice.polini@farm.unipi.it (B.P.); clementina.manera@unipi.it (C.M.); maria.digiacomo@unipi.it (M.D.); ja.espositosalsano@student.unisi.it (J.E.S.); giulio.poli@unipi.it (G.P.); tiziano.tuccinardi@unipi.it (T.T.); marco.macchia@unipi.it (M.M.); paola.nieri@unipi.it (P.N.); 2 Interdepartmental Research Center “Nutraceuticals and Food for Health” University of Pisa, 56124 Pisa, Italy; 3 National Research Council (CNR) Institute of Clinical Physiology (IFC), 73100 Lecce, Italy; egeria.scoditti@ifc.cnr.it (E.S.); marika.massaro@ifc.cnr.it (M.M.); maria.carluccio@ifc.cnr.it (M.A.C.); 4 Doctoral School in Life Sciences, University of Siena, 53100 Siena, Italy; 5 Molecular Medicine for Neurodegenerative and Neuromuscular Diseases Unit, IRCCS Fondazione Stella Maris, Calambrone, 56128 Pisa, Italy; stefanodoccini@gmail.com (S.D.); filippo3364@gmail.com (F.M.S.); 6 Division of Pediatric Endocrinology, Diabetes and Obesity, Department of Pediatrics and Adolescent Medicine, University of Ulm, 89075 Ulm, Germany; Martin.Wabitsch@uniklinik-ulm.de; 7 Cardiology Division, Pisa University Hospital, 56126 Pisa, Italy; raffaele.decaterina@unipi.it

**Keywords:** adipocyte, exosome, extra-virgin olive oil, gene expression, inflammation, obesity, oleocanthal, oleacein, miRNA, NF-κB

## Abstract

Inflammation of the adipose tissue plays an important role in the development of several chronic diseases associated with obesity. Polyphenols of extra virgin olive oil (EVOO), such as the secoiridoids oleocanthal (OC) and oleacein (OA), have many nutraceutical proprieties. However, their roles in obesity-associated adipocyte inflammation, the NF-κB pathway and related sub-networks have not been fully elucidated. Here, we investigated impact of OC and OA on the activation of NF-κB and the expression of molecules associated with inflammatory and dysmetabolic responses. To this aim, fully differentiated Simpson-Golabi-Behmel syndrome (SGBS) adipocytes were pre-treated with OC or OA before stimulation with TNF-α. EVOO polyphenols significantly reduced the expression of genes implicated in adipocyte inflammation (IL-1β, COX-2), angiogenesis (VEGF/KDR, MMP-2), oxidative stress (NADPH oxidase), antioxidant enzymes (SOD and GPX), leukocytes chemotaxis and infiltration (MCP-1, CXCL-10, MCS-F), and improved the expression of the anti-inflammatory/metabolic effector PPARγ. Accordingly, miR-155-5p, miR-34a-5p and let-7c-5p, tightly connected with the NF-κB pathway, were deregulated by TNF-α in both cells and exosomes. The miRNA modulation and NF-κB activation by TNF-α was significantly counteracted by EVOO polyphenols. Computational studies suggested a potential direct interaction between OC and NF-κB at the basis of its activity. This study demonstrates that OC and OA counteract adipocyte inflammation attenuating NF-κB activation. Therefore, these compounds could be novel dietary tools for the prevention of inflammatory diseases associated with obesity.

## 1. Introduction

Inflammation of adipose tissue (AT) plays an important role in the development of many chronic diseases associated with obesity [1]. Both animal and clinical investigations suggest that inflammation and dysfunction of AT in obesity, resulting in the aberrant production of inflammatory mediators, are key processes linking obesity to comorbidities, including insulin resistance and type 2 diabetes, cardiovascular and respiratory diseases, osteoarthritis, and cancer [2].

Until recently, adipocytes, the main cellular components of AT, were mainly considered as an energy depot, but there is now clear evidence pointing to an active role of adipocytes as endocrine cells producing hormones, growth factors, cytokines, chemokines, collectively called adipo(cyto)kines. These proteins can act in an autocrine, paracrine or endocrine fashion to control various functions, including energy homeostasis and metabolism in the AT itself, the liver, the skeletal muscle and the vasculature [3]. Altered adipocyte function, as it occurs in obesity and overweight, especially increased intra-abdominal adiposity, is accompanied by tissue remodeling and oxidative stress, and deleteriously changes the expression and production of pro- and anti-inflammatory, metabolic and proliferative factors, which leads to a tissue and systemic low-grade proinflammatory state and the development of obesity comorbidities [3]. Therefore, targeting adipocyte dysfunction and inflammation is an attractive perspective for the treatment of obesity-related metabolic and vascular diseases. With the increase of AT mass, the production of insulin-sensitizing and anti-inflammatory adipokines, such as adiponectin, drops. Contrarily, enlarged adipocytes start producing adipokines, such as chemokines and cytokines, affecting the AT infiltration of immune cells, mainly monocytes/macrophages, granulocytes and lymphocytes. In particular, a key event in the induction of obesity-induced AT inflammation and development of insulin resistance appears to be the polarization of AT macrophages from an anti-inflammatory M2 phenotype to a pro-inflammatory M1 phenotype [4]. During obesity, signals mostly from hypertrophic and dysfunctional adipocytes drive M2-to-M1 transition of AT macrophages, which then become a major source of pro-inflammatory factors, such as tumor necrosis factor (TNF)-α and interleukin (IL)-1β, thus amplifying adipocyte dysfunction and recruiting and activating more pro-inflammatory cells in a vicious cycle, and ultimately contributing to local and systemic chronic low-grade inflammation and insulin resistance [1].

A main player in inflammation is nuclear factor-κB (NF-κB). NF-κB is a family of inducible transcription factors, which regulate the expression of genes, such as chemokines, adhesion molecules, cytokines, involved in processes of immune and inflammatory responses [5], and has been implicated in the initiation and progression of metabolic diseases, connecting inflammation and dysmetabolism in the AT [6]. In resting cells, NF-κB is sequestered in the cytosol bound to Inhibitor of κB (IκB) proteins, which prevents the nuclear localization and transcriptional function of NF-κB. After stimulation with cytokines, microbial factors, fatty acids and other stimuli such as those in obesity, the IκB kinase (IKK) complex containing two catalytic subunits (IKKα and IKKβ) is activated, which triggers the phosphorylation and degradation of IκB, and promotes NF-κB translocation to the nucleus and the transcription of target genes. Either genetic deficiency or chemical inhibition of components of the NF-κB pathway including IKKβ has been found to significantly decrease obesity-induced insulin resistance in humans and mice [7,8,9]. Indeed, stimulation of the NF-κB pathway has been reported to induce insulin resistance through both direct serine phosphorylation of insulin receptor substrate (IRS)-1/-2 mediated by IKKβ as well as the upregulation of pro-inflammatory cytokines, which in turn activate serine kinases such as IKKβ and mitogen-activated protein kinase (MAPK) causing IRS inhibition, and induce other inflammation-related negative regulators of insulin signaling [10]. Therefore, targeting NF-κB-driven inflammation may translate into beneficial modulatory effects on metabolic diseases. 

Other players increasingly recognized in obesity-associated inflammation and comorbidities are microRNAs (miRNAs), small non-coding RNAs that exert regulatory effects on gene expression by degrading complementary mRNA targets and inhibiting translation [11]. These miRNAs play essential roles in many biological processes, including survival, proliferation, differentiation, apoptosis, metabolism, and immunity. Recently, several miRNAs have emerged as involved in pathways related to obesity such as adipocyte differentiation, adipokine expression, glucose and lipid metabolism, insulin signalling, oxidative stress, and inflammation [12,13]. Such miRNAs can be secreted by adipocytes, and transferred by membrane-bound extracellular vesicles, namely exosomes (from intracellular endosomes and typically 30–100 nm in diameter) and microvesicles (from plasma membrane and 0.1–1 μm in diameter) [14], to neighbouring and distant cells, where they may exert both local and systemic regulatory effects, including immune cell activation, insulin resistance, inflammation and fibrosis [15]. Both the AT and circulating miRNAs are deregulated in human obesity [16,17,18]. The miRNA profiling in adipocytes and their supernatants in inflammatory conditions has revealed sustained and concordant changes in the expression of a range of miRNAs related to adipogenesis, oxidative stress, the insulin pathway, and inflammation [13]. 

Among AT-related miRNAs, miR-155 [19,20], miR-34a [16,21], and let-7 [22,23] appear to be involved in inflammatory responses and linked to the NF-κB pathway [24,25,26,27,28,29] Consequently, the ability to control NF-κB and its sub-network activation could contribute to modulating pathogenic processes of various inflammatory diseases, also connected to adipocytes inflammation.

Accumulating evidence suggests that extra virgin olive oil (EVOO), an integral ingredient of the Mediterranean diet, exerts health benefits that include the modulation of inflammatory and immune responses, the prevention of cancers and the reduction in the risk of coronary heart disease and metabolic disease [30]. Contributory roles have been ascribed to its main components including monounsaturated fatty acids (particularly oleic acid) and polyphenols. In particular, polyphenols include phenolic alcohols such as hydroxytyrosol (3,4-dihydroxyphenylethanol), tyrosol (*p*-hydroxyphenylethanol), as well as secoiridoids that contain either elenolic acid or elenolic acid derivatives in their molecular structure, such as the aglycone of oleuropein, the aglycone of ligstroside, and their respective decarboxylated dialdehyde derivatives oleocanthal (*p*-hydroxyphenylethanolelenolic acid dialdehyde or decarboxymethyl ligstroside aglycone, OC) and oleacein (3,4-dihydroxyphenylethanolelenolic acid dialdehyde, OA) [31]. EVOO is a rich source of phenolic compounds (40–1000 mg/kg), which not only are strong antioxidants and radical scavengers both in vitro and in vivo, but also possess other potent biological activities, including anti-inflammatory, immunomodulatory, hypotensive, anti-microbial and metabolic effects, that could partially account for the observed health effects of the Mediterranean diet [32].

Among these phenolic constituents, two key secoiridoids, OC and OA, are attracting clinical and nutritional interest due to their proved bioactivity. Early research conducted by Beauchamp and colleagues demonstrated that OC inhibits the pro-inflammatory cyclooxygenase (COX) enzymes in a dose-dependent manner, mimicking the anti-inflammatory action exerted by ibuprofen [33]. Subsequent investigations demonstrated OC efficacy against inflammatory diseases, including joint degenerative disease, neurodegenerative disease and specific cancers [34,35]. The oleuropein derivative OA is another recently emerged, potentially healthful polyphenol, exerting anti-inflammatory, antioxidant and vasculoprotective effects [36,37].

Polyphenols from several food sources have been increasingly found to favorably affect AT physiology [38], and their modes of action include interaction with cell signaling pathways, modulation of transcription factors activity and gene expression, but also the post-transcriptional regulation of genes via modulation of miRNAs expression [39,40,41]. 

Data concerning the action of EVOO polyphenols in the context of human obesity-related AT inflammation and dysfunction are scarce, and mainly regard oleuropein and its derivative hydroxytyrosol, demonstrating potential anti-obesity and anti-diabetic effect [42,43,44,45,46]. Recently, beneficial properties of in vivo administration of OA have been reported, showing protective effects against weight gain, insulin resistance, liver steatosis, and lipid metabolism in high fat diet-fed mice [47]. Data on the effect of OC and OA on obesity-associated inflammatory responses in adipocytes are lacking. In the light of their anti-inflammatory action, we hypothesized an effect of OC and OA on adipocyte inflammatory dysfunction as a mechanism underlying the beneficial effects of EVOO in the prevention of cardio-metabolic risk. We here assessed the effect of OC and OA on the expression of inflammation-related genes (transcripts) and miRNAs in human adipocytes, under an obesity-mimicking inflammation induced by the cytokine TNF-α.

## 2. Materials and Methods

### 2.1. Extraction, Purification, and Characterization of OC and OA

The extraction and purification of OC and OA were performed using a previously reported procedure [35,48].

### 2.2. Cell Culture and Treatments

As a physiologically relevant cell model system resembling human AT, we used human Simpson-Golabi-Behmel syndrome (SGBS) preadipocytes [49]. These were a generous gift of our coworker Prof. Martin Wabitsch, and were cultured and differentiated into mature adipocytes, as previously described [45]. Briefly, SGBS cells were maintained in Dulbecco’s Modified Eagle Medium (DMEM)/F12 containing 10% fetal bovine serum (FBS) and 1% penicillin/streptomycin, 33 µmol/L biotin and 17 µmol/L pantothenate. For experimental purposes, cells were plated and allowed to reach confluence before the addition of serum-free differentiation medium (DMEM/F12 with 25 nmol/L dexamethasone, 250 µmol/L 3-isobutyl-1-methylxanthine, 2 µmol/L rosiglitazone, 0.01 mg/mL human transferrin, 20 nmol/L insulin, 100 nmol/L cortisol, 0.2 nmol/L triiodothyronine, 33 µmol/L biotin, and 17 µmol/L pantothenate) for 4 days. Cell medium was then changed to an adipogenic medium (DMEM/F12 with 0.01 mg/mL human transferrin, 20 nmol/L insulin, 100 nmol/L cortisol, 0.2 nmol/L triiodothyronine, biotin, and pantothenate) for further 10 days. On day 15, >90% of these cells undergo complete differentiation into mature adipocytes, as assessed using Oil Red-O lipid staining and the expression of adipocyte-specific markers. 

AT inflammation has been mimicked by incubating fully differentiated SGBS cells with medium supplemented with the proinflammatory cytokine TNF-α at 10 ng/mL during 18 h. Unstimulated controls were adipocytes incubated in medium without TNF-α. For polyphenols treatment, SGBS cells were incubated with 25 µmol/L OC or OA for 6 h before stimulation with TNF-α. After treatments, cell culture medium were collected, centrifuged to remove cell debris and stored at −80 °C until analysis.

### 2.3. Cell Viability

Cell viability was determined by the 3(4,5dimethylthiazol2yl) 2,5diphenyltetrazolium bromide (MTT) assay, a commonly used method to evaluate cell survival, based on the ability of viable cells to convert MTT, a soluble tetrazolium salt, into an insoluble formazan precipitate, which is quantitated spectrophotometrically. Briefly, after the pertinent treatment, cells were incubated with MTT (0.5 mg/mL) for 4 h, and the formazan products were then dissolved by isopropanol. Absorbance was measured at 490 nm on a microplate reader.

### 2.4. Measurement of MCP-1 in Culture Media

Levels of monocyte chemoattractant protein (MCP-1) in the culture medium were determined using a human MCP-1/CCL2 PicoKine™ ELISA kit (Boster, Pleasanton, CA, USA) according to the manufacturer’s instructions. MCP-1 concentration was calculated from the standard curve, normalized to total protein content, and expressed as percent of TNF-α.

### 2.5. RNA Isolation and Real-Time Quantitative Polymerase Chain Reaction

Total RNA was isolated by using the TRIzol reagent (Invitrogen, Carlsbad, CA, USA) according to the manufacturer’s protocol. For real-time quantitative polymerase chain reaction (qPCR), total RNA (2 µg) was converted into first-strand cDNA by using the High Capacity cDNA Reverse Transcription Kit (Applied Biosystems, Monza, Italy). The qPCR was performed in a CFX Connect Real-Time PCR Detection System (Bio-Rad Laboratories, Milan, Italy) by using primer sequences or Taqman Gene Expression Assays for the indicated markers. All reactions were done in triplicate, and the amount of mRNA calculated by the comparative critical threshold (C_T_) method. To account for possible variations related to cDNA input or the presence of PCR inhibitors, the endogenous reference gene ribosomal 18S was simultaneously quantified for each sample, and data normalized accordingly. Results are expressed as fold increase relative to unstimulated control (made = 1).

### 2.6. Preparation of Nuclear Extracts and Measurement of NF-κB p65 DNA Binding Activity

Nuclear proteins were extracted using the “Nuclear Extract Kit” (Active Motif, Carlsbad, CA, USA) according to the manufacturer’s protocol. The activation of NF-κB was assayed using the enzyme linked immunosorbent assays (ELISA)-based TransAM NF-κB p65 kit (Active Motif), following the manufacturer’s protocol. Briefly, the NF-κB TransAM kit contains a 96-well plate with immobilized oligonucleotides encoding an NF-κB consensus site (5′-GGGACTTTCC-3′). The active form of NF-κB contained in the nuclear cell extracts specifically binds to the oligonucleotide. The primary antibody used to detect NF-κB recognizes an epitope on p65 that is accessible only when NF-κB is activated and bound to its target DNA sequence. A horseradish peroxidase-conjugated secondary antibody provides a sensitive colorimetric readout that is quantified by spectrophotometry at 450 nm, with a reference wavelength set to 655 nm. Data were expressed as percent of unstimulated control.

### 2.7. Exosome Isolation from Cell Culture Supernatants

Exosomes were isolated from cell culture conditioned supernatants using the protocol published by Lobb et al. [50]. Conditioned culture media were harvested from adipocytes and centrifuged at 300× *g* at 4 °C for 10 min to remove detached cells. Then, supernatants were filtered through 0.22 µm filters (Merck Millipore, Darmstadt, Germany) to remove contaminating apoptotic bodies, microvesicles and cell debris. Clarified conditioned culture media were then centrifuged in a Sorvall^TM^ MTX 150 micro-ultracentrifuge (Thermo Scientific, Waltham, MA, USA) at 100,000× *g* at 4 °C for 90 min to pellet exosomes. The supernatants were carefully removed, and pellets containing exosomes were resuspended in 1 mL of ice-cold PBS. A second round of ultracentrifugation under the same condition was carried out, and the resulting exosome pellets resuspended in 200 µL of PBS.

### 2.8. Evaluation of miRNA Expression

The miRNeasy Mini Kit (Qiagen, Hilden, Germany) was used for purification and extraction of miRNAs from exosomes isolated from cell culture conditioned supernatants or adipocytes. The retro-transcription of extracted miRNAs was performed by using the miScript Reverse Transcription Kit (Qiagen) [51]. The cDNA obtained was diluted 1:3 in RNase-free water from adipocytes, while the exosome-cDNA was used without dilution. The qPCR experiments were performed by miScript SYBR-Green PCR kit (Qiagen), as previously reported [52]. Signals were detected on the MiniOpticon CFX 48 real-time PCR Detection System (Bio-Rad, Hercules, CA, USA). MiScript Primer Assays specific for hsa-miR-34a-5p (MIMAT0000255), hsa-miR-155-5p (MIMAT0000646) and hsa-let-7c-5p (MIMAT0000064) and hsa-SNORD6 were obtained from Qiagen. The expression of miRNAs was calculated using in the comparative cycle threshold (Ct) method and normalized to the expression of housekeeping genes SNORD6 for adipocyte-derived miRNAs, and *Caenorhabditis elegans* miR-39 (Cel-miR-39) for exosome-derived miRs (exo-miRs).

### 2.9. miRNA Target Prediction and Pathway Analysis

Predicted targets from TargetScan (Human version 7.2) were subjected to computational analysis with the Database for Annotation, Visualization and Integrated Discovery (DAVID) (version 6.8) [53] to identify biological processes associated to the miRNAs modulated by EVOO polyphenols. 

### 2.10. Molecular Docking

The structure of OC was constructed with Maestro [54] and then subjected to energy minimization into a water environment performed with Macromodel [55], using the generalized Born/surface area model, the conjugate gradient algorithm, the MMFFs force field and a distance-dependent dielectric constant of 1.0, until a convergence value of 0.05 kcal/(Å·mol) was reached. OC was docked into the X-ray structure of the human NF-κB p50/p65 heterodimer obtained from the crystal structure of the partial interferon-β enhancesome, which contained the DNA-binding domains of IRF-3, IRF-7 and NF-κB bound to one half of the enhancer (PDB code 2O61) [56]. Docking studies were performed with AUTODOCK4.2 [57], using a robust protocol already employed in previous studies [58,59]. Prior to docking calculations, the DNA fragment as well as the peptide chains corresponding to the DNA-binding domains of IRF-3 and IRF-7 were removed, thus leaving the NF-κB p50/p65 heterodimer as the receptor. AUTODOCK TOOLS [60] were employed to define the torsion angles in the ligand, to add the solvent model and to assign partial atomic charges (Gasteiger for the ligands and Kollman for the receptors). Since we did not want to bias the docking protocol toward the generation of poses located in specific protein sites, OC was docked into a grid box of 70 points in the x, y and z directions, comprising all major contact points between NF-κB p50/p65 heterodimer and the bound DNA fragment, in the reference X-ray structure. The energetic map calculations were carried out by using a grid spacing of 0.375 Å and a distance-dependent function of the dielectric constant. The ligand was subjected to 200 runs of the AUTODOCK search using the Lamarckian genetic algorithm with 10,000,000 steps of energy evaluation; the number of individuals in the initial population was set to 500, and a maximum of 10′000′000 generations were simulated during each docking run. All other settings were left as their defaults, and the final solutions produced were clustered using a 2.0 Å RMSD cutoff. The clusters of solutions with a population higher than 5%, i.e., including more than 5% of all the generated docking poses, were taken into account, for a total of 5 different clusters. The same docking procedure was used to dock OC into the crystal structure of the human IκB kinase (IKKβ) in complex with the staurosporine analog K252a (PDB code 4KIK) [61]. Prior to docking calculations, the peptide chains not included in the protein kinase domain were removed. OC was docked into the catalytic site of the protein, using a grid box of 50 points in the x, y and z directions centered on the bound ligand. The best docking pose was taken into account.

### 2.11. Molecular Dynamic Simulations

All molecular dynamic (MD) simulations were performed with AMBER 16 [62] employing the ff14SB force field following a validated protocol already used in pose-prediction studies [63,64]. General Amber Force Field (GAFF) parameters were assigned to the ligands, while partial charges were calculated using the AM1-BCC method as implemented in the Antechamber suite of AMBER 16. The five different OC-p50/p65 complexes and the OC-IKKβ complex were placed in a parallelepiped water box using the TIP3P explicit solvent model and solvated with a 10 Å water cap, while chloride ions were added as counterions to neutralize the systems. Prior to MD simulations, the solvated complexes were energy-minimized through 5000 steps of steepest descent, followed by conjugated gradient, using Particle mesh Ewald electrostatics, periodic boundary conditions and a cutoff of 10 Å for the non-bonded interactions. All bonds involving hydrogen atoms were kept rigid using the SHAKE algorithm, so that a time step of 2.0 fs could be used for the MD. The minimized systems were subjected to an initial MD step of 0.5 ns performed with constant-volume periodic boundary conditions, during which the temperature of the system was raised from 0 to 300 K. A following constant-pressure periodic boundary MD was then performed for 3 ns to allow the equilibration of the system; in this step the temperature was kept at 300 K using the Langevin thermostat. In the final simulation step, 26.5 ns of constant-pressure and temperature MD were performed, for a total simulation time of 30 ns. During all three MD steps, a harmonic potential of 10 kcal/(mol·Å^2^) was applied to the protein α-carbons.

### 2.12. Binding Energy Evaluations

Ligand-protein binding energy evaluations were performed as already reported [65,66], using AMBER 16. The trajectories relative to the last 20 ns of MD simulations generated for the OC-p50/p65 complexes were employed for the calculations, for a total of 200 snapshots (at time intervals of 100 ps). The gas and water phases of the systems were represented using dielectric constants of 1 and 80, respectively. Van der Waals, electrostatic, and internal interactions were calculated with the SANDER module of AMBER 16. Polar energies were calculated using the Poisson-Boltzmann method with the MM-PBSA module of AMBER 16, while nonpolar energies were estimated using the MOLSURF program.

### 2.13. Statistical Analysis

Results are expressed as means ± SD of at least three independent experiments performed in triplicate. miRNA expression was presented as box-plots. We used the Student’s *t* test for comparing means between control group and compound-treated group. We performed multiple comparisons by one-way analysis of variance (ANOVA). A *p* level <0.05 was considered statistically significant. 

## 3. Results

### 3.1. OC and OA Attenuate TNF-α–Mediated Inflammatory Gene Expression in Human Adipocytes

**Figure 1:**
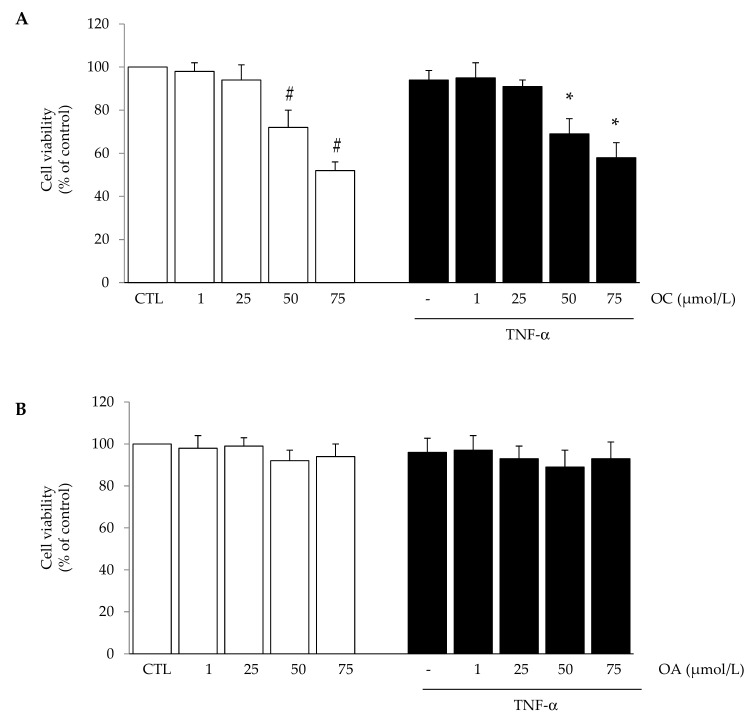
The effect of oleocanthal (OC) and oleacein (OA) treatment on cell viability. Simpson-Golabi-Behmel syndrome (SGBS) adipocytes were treated with OC (**A**) or OA (**B**) for 6 h at the concentrations indicated, and then either treated with 10 ng/mL TNFα (black filled bars), or left untreated (open white bars), for 18 h. Cell viability was assessed by the 3(4,5dimethylthiazolyl)-2,5-diphenyl-tetrazolium bromide (MTT) assay, and expressed as percent of unstimulated control (CTL). Data are means ± SD (n = 3). # *p* < 0.05 versus CTL. * *p* < 0.05 versus TNF-α alone.

Preliminary experiments were conducted aiming at testing the effects of OC and OA on cell viability and finding an effective anti-inflammatory concentration to be used for subsequent experiments. With the exception of OC which reduced cell viability at concentrations ≥50 µmol/L after 24 h, treatment of SGBS cells with OC (1–25 µmol/L) or OA (1–75 µmol/L) in the absence or presence of TNF-α for 24 h did not affect cell viability, as assessed by the MTT test (Figure 1A,B), morphological observation, protein assay and Trypan blue exclusion (data not shown). 

**Figure 2:**
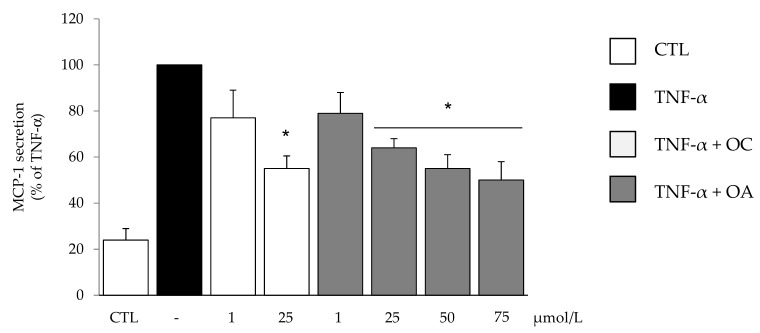
The effect of OC and OA on monocyte chemoattractant protein (MCP-1) protein release in the culture medium. SGBS cells were pretreated (6 h) with either OC (1–25 µmol/L) or OA (1–75 µmol/L) before 10 ng/mL TNFα stimulation for 18 h. MCP-1 in the culture medium was determined by ELISA, and expressed as percent of TNF-α stimulation. Data are means ± SD (n = 3). * *p* < 0.05 versus TNF-α alone.

A subsequent dose-response study was performed to find OC and OA concentrations effective in attenuating the release of a prototypical inflammatory adipokine, MCP-1, the potent chemoattractant specific for monocytes and macrophages, in the culture medium. TNF-α-stimulated protein release of MCP-1 was reduced by both OC and OA in a concentration-dependent manner, with a significant effect starting at 25 µmol/L (Figure 2). 

**Figure 3:**
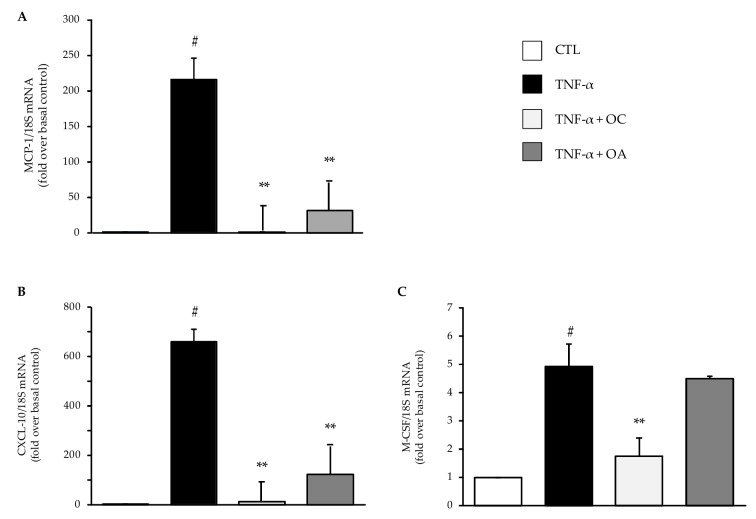
Modulation by OC and OA of mRNA expression levels of chemokines associated with adipocyte inflammation. SGBS adipocytes were pretreated with OC or OA 25 µmol/L (6 h) and then treated with 10 ng/mL TNF-α for 18 h. Total RNA was extracted from cells, and mRNA levels of the monocyte chemoattractant protein (MCP-1) (**A**), C-X-C Motif Ligand 10 (CXCL-10) (**B**), and macrophage colony-stimulating factor (M-CSF) (**C**) were measured by qPCR using specific primers and probes and normalized to 18S RNA. Data (means ± SD, n = 3) are expressed as fold induction over unstimulated control (CTL). # *p* < 0.05 versus CTL. ** *p* < 0.01 versus TNF-α alone.

Thereafter, based on these combined results, we studied the effects of OC and OA at 25 µmol/L on mRNA expression levels of several adipokines involved in AT inflammation, insulin resistance, oxidative stress and tissue remodeling. Cell pre-treatment with OC or OA before stimulation with TNF-α significantly prevented the TNF-α-induced upregulation of the mRNA levels of the chemokines MCP-1 (Figure 3A), C-X-C Motif Ligand 10 (CXCL-10) (Figure 3B), and, only for OC, macrophage colony-stimulating factor (M-CSF) (Figure 3C).

**Figure 4:**
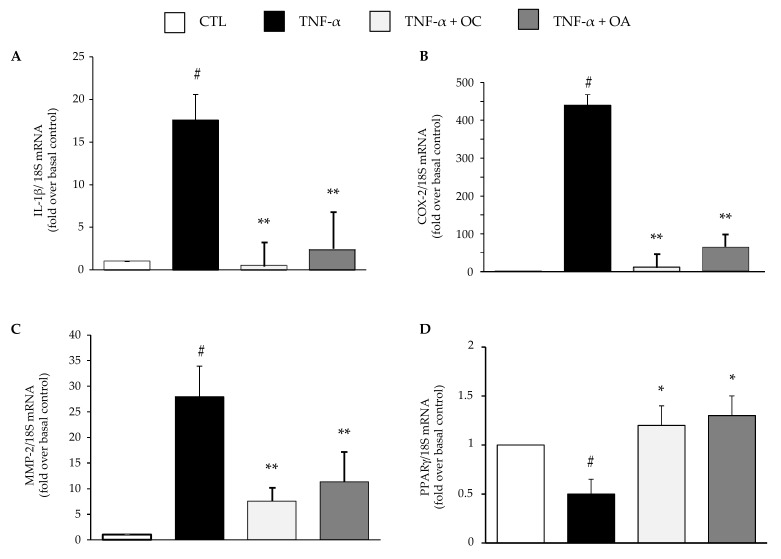
Modulation by OC and OA of mRNA expression levels of cytokines, inflammatory and remodeling enzymes, and metabolic transcriptional regulator associated with adipocyte inflammation. SGBS adipocytes were pretreated with OC or OA 25 µmol/L (6 h) and then treated with 10 ng/mL TNF-α for 18 h. Total RNA was extracted from cells, and mRNA levels of interleukin-1β (IL-1β) (**A**), cyclooxygenase-2 (COX-2) (**B**), metalloproteinase-2 (MMP-2) (**C**), and peroxisome proliferator-activated receptor-γ (PPARγ) (**D**) were measured by qPCR using specific primers and probes and normalized to 18S RNA. Data (means ± SD, n = 3) are expressed as fold induction over unstimulated control (CTL). # *p* < 0.05 versus CTL. * *p* < 0.05 versus TNF-α alone. ** *p* < 0.01 versus TNF-α alone.

Both compounds also attenuated the TNF-α-stimulated increase in the mRNA expression of the cytokine interleukin(IL)-1β (Figure 4A), the pro-inflammatory enzyme COX-2 (Figure 4B), and the matrix-degrading enzyme metalloproteinase(MMP)-2 (Figure 4C). Contrarily, OC and OA significantly prevented the TNF-α-induced downregulation of peroxisome proliferator-activated receptor-γ (PPARγ) mRNA levels, a transcription factor involved in the control of energy metabolism and inflammation in AT (Figure 4D). 

**Figure 5:**
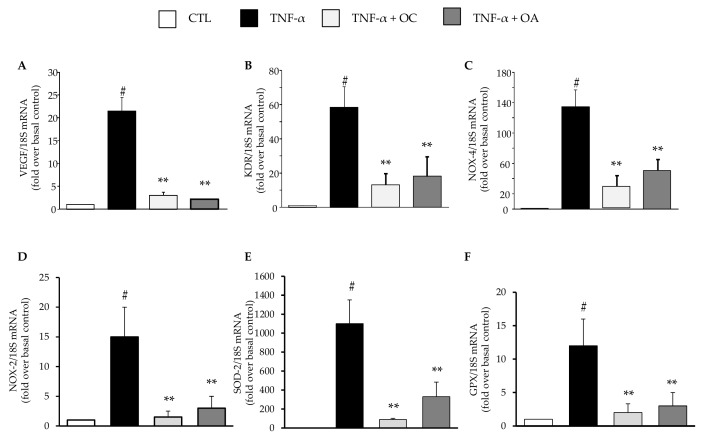
Modulation by OC and OA of mRNA expression levels of vascular endothelial growth factor (VEGF) and its receptor receptor kinase insert domain receptor (KDR), the pro-oxidant enzyme NADPH oxidase-4 (NOX)-4 and NOX-2, and the antioxidant enzymes superoxide dismutase (SOD)-2 and glutathione peroxidase (GPX). SGBS adipocytes were pretreated with OC or OA 25 µmol/L (6 h) and then treated with 10 ng/mL TNF-α for 18 h. Total RNA was extracted from cells, and mRNA levels of VEGF (**A**), KDR (**B**), NOX-4 (**C**), NOX-2 (**D**), SOD-2 (**E**) and GPX (**F**) were measured by qPCR using specific primers and probes and normalized to 18S RNA. Data (means ± SD, n = 3) are expressed as fold induction over unstimulated control (CTL). # *p* < 0.05 versus CTL. ** *p* < 0.01 versus TNF-α alone.

Similarly, a significant inhibitory effect by OC and OA was also observed on the mRNA levels of the pro-angiogenic vascular endothelial growth factor (VEGF) (Figure 5A) and its receptor kinase insert domain receptor (KDR or VEGFR-2) (Figure 5B), as well as the pro-oxidant enzyme NADPH oxidase (NOX)-4 and NOX-2 (Figure 5C,D). OC and OA also attenuated the increase of superoxide dismutase (SOD)-2 and glutathione peroxidase (GPX) mRNA levels in response to TNF-α (Figure 5E,F). OC exhibited a more potent inhibitory effect compared with OA for most of the investigated genes. 

### 3.2. OC and OA Modulate TNF-α-Induced Inflammation-Linked miRNAs Expression in Adipocytes and Adipocyte-Derived Exosomes

To evaluate the potential modulatory effect of EVOO polyphenols on miRNA expression related to inflammation, SGBS adipocytes were pre-incubated with OC or OA for 6 h and then stimulated with TNF-α for 18 h, and levels of miRNAs extracted both from adipocytes and exosomes released in the culture medium (exo-miRNAs) were analysed by qPCR.

**Figure 6:**
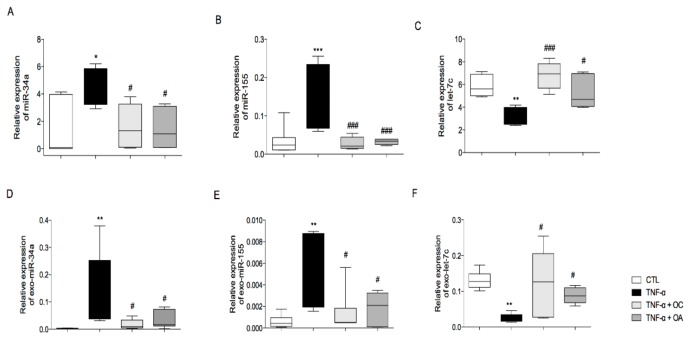
Modulation by OC and OA of TNF-α-induced miRNA expression in adipocytes and in exosomes isolated from conditioned media. SGBS cells were pretreated with OC or OA 25 µmol/L (6 h) and then stimulated with 10 ng/mL TNF-α for 18 h. Total miRNAs were extracted from cells, and miR-34a-5p (**A**), miR-155-5p (**B**) and let-7c-5p levels (**C**) were measured by qPCR, analysed using the Ct method and normalized to SNORD6 levels. Total miRNAs were extracted from exosomes, and exo-miR-34a (**D**), exo-miR-155 (**E**) and exo-let-7c (**F**) levels were measured by qPCR, analyzed using the Ct method and normalized to Cel-miR-39. Bars represent means ± SD (n = 3). * *p* < 0.05 versus CTL. ** *p* < 0.01 versus CTL. *** *p* < 0.001 versus CTL. # *p* < 0.05 versus TNF-α. ### *p* < 0.001 versus TNF-α.

miR-34a expression was significantly up-regulated in adipocytes and exosomes under TNF-α treatment, as compared to levels found in untreated cells. Pre-treatment with OC or OA significanly counteracted the high expression of miR-34 (Figure 6A,D). 

The same trend was observed for miR-155 in adipocytes and exosomes. In particular, treatment with TNF-α induced a significant increase in the levels of miR-155-5p, but this did not occur with pretreatment with OC or OA 

Finally, let-7c expression was significantly downregulated by TNF-α in adipocytes and exosomes, as compared to levels found in untreated cells. In cells and exosomes, pretreatment with OC or OA prevented this decreased expression of let-7c (Figure 6C,F). 

### 3.3. Biological Processes Associated to miRNAs Modulated by Polyphenols in Inflamed Adipocytes

By using the DAVID tool, we performed the analysis of biological processes associated with genes potently altered by the OC- and OA-modulated miRNAs and exo-miRNAs in inflammatory condition: 1242 mRNA genes were associated with the two dowregulated miRNAs, i.e., miR-34a-5p, miR-155-5p, and 1207 mRNA genes were associated with the upregulated miR, i.e., let-7c-5p.

Gene ontology (GO) analysis revealed that miR-155 and miR-34a are involved in 310 biological process (BP). Among those, both miRNAs were significantly associated with 189 BP (Table S1), such as ‘positive regulation of NF-κB transcription factor activity’ (25 genes, *p* = 0.0009), ‘angiogenesis‘ (26 genes, *p* = 0.0057), ‘response to glucose’ (11 genes, *p* = 0.0120), ‘cellular response to vascular endothelial growth factor stimulus’ (6 genes, *p* = 0.0150), ‘interleukin-6-mediated signaling pathway’ (four genes, *p* = 0.0170), ‘positive chemotaxis’ (seven genes, *p* = 0.0240), and ‘regulation of cell size’ (five genes, *p* = 0.0380). 

Regarding let-7c, GO analysis revealed its overall involvement in 192 BP and significant association with 161 BP (Table S2), such as ‘cellular response to cytokine stimulus’ (five genes, *p* = 0.044) and ‘regulation of cytokine biosynthetic process’ (three genes, *p* = 0.045). 

**Figure 7:**
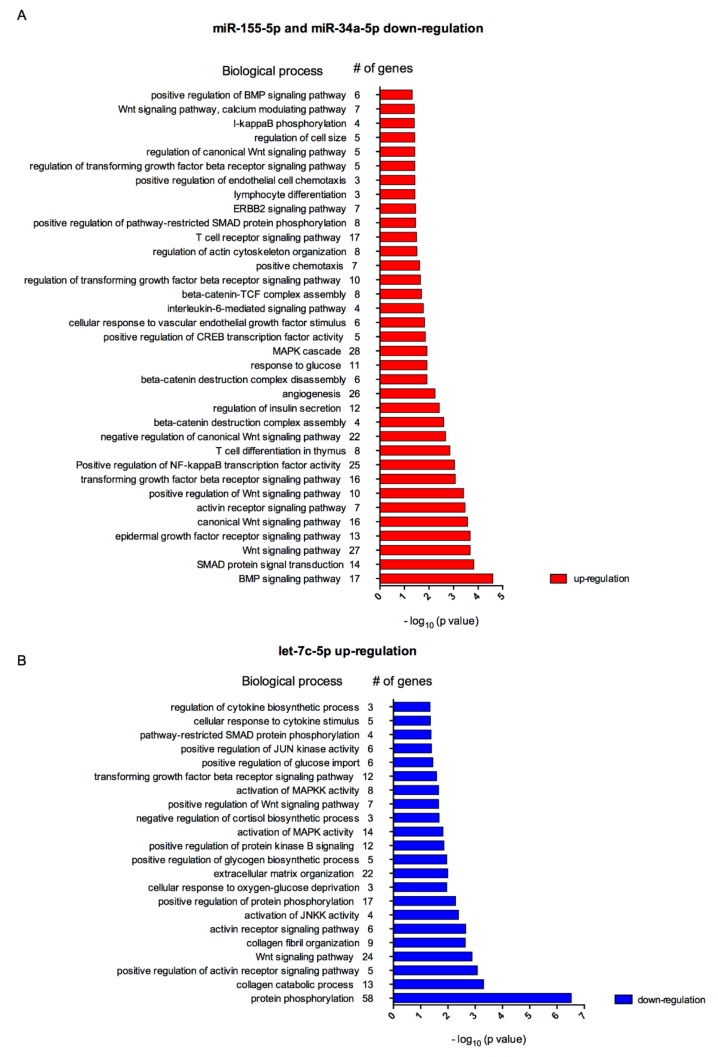
Biological processes associated to target genes of miRNAs regulated by OC and OA, i.e., down-regulation of miR-155-5p and miR-34a-5p (**A**) and up-regulation of let-7c-5p (**B**) in human adipocytes.

As reported in Figure 7A,B, various BP modulated by these miRNAs were connected with adipocyte inflammatory processes, such as adipocytes expansion, recruitment of macrophages, adipocytokines cocktail secretion, tissue remodelling and glucose homeostasis [67].

### 3.4. NF-κB Inhibition by OC and OA in Human Adipocytes

#### 3.4.1. OC and OC Attenuate TNF-α-Induced Activation of NF-κB

**Figure 8:**
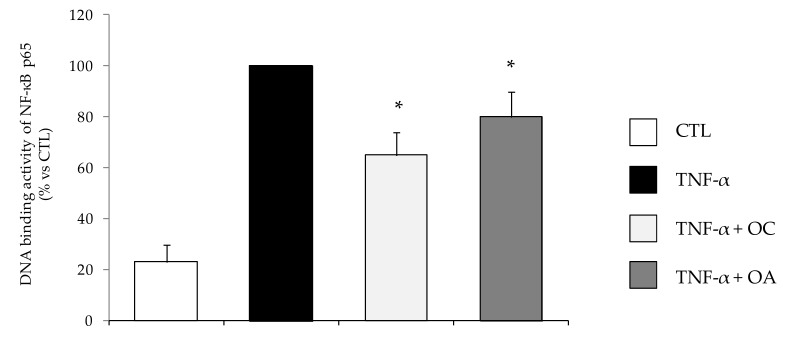
Modulation by OC and OA of NF-κB activation. SGBS adipocytes were pretreated with OC or OA 25 µmol/L (6 h) and then treated with 10 ng/mL TNF-α for 1 h. Then, nuclear proteins were extracted and assessed by ELISA to measure the DNA binding activity of the p65 NF-κB subunit. Data (means ± SD, n = 3) are expressed as percent of TNF-α. CTL = unstimulated control. * *p* < 0.05 versus TNF-α alone.

Being the transcription factor NF-κB a master regulator of the expression of genes and miRNAs, including those here tested, orchestrating inflammatory responses [6,27], we examined the effect of OC and OA on the ability of TNF-α to activate NF-κB in human adipocytes. To this aim, SGBS cells were pretreated with OC or OA and then stimulated with TNF-α for 1 h to induce NF-κB activation and nuclear translocation. The capacity of the p65 subunit of NF-κB to bind to the DNA consensus site, which may correlate positively with the activation of NF-κB, was quantified in nuclear extracts by an ELISA-based method. In this assay, the active form of NF-κB contained in nuclear extract specifically binds to the NF-κB consensus oligonucleotide immobilized onto a 96-well-plate. A primary antibody recognizes an epitope of p65, which is accessible only when NF-κB is activated and bound to its target DNA. A horseradish peroxidase-conjugated secondary antibody allows for spectrophotometric quantification of the amount of p65 subunit bound. Expectedly, as shown in the Figure 8, TNF-α induced a marked increase in the DNA binding activity of the p65 NF-κB subunit. Cell exposure to OC or OA reduced, by about 35% and 20%, respectively, NF-κB activation in response to TNF-α.

#### 3.4.2. Molecular Modeling Studies of OC Interaction with NF-κB

In order to rationalize the experimental results obtained, molecular modeling studies, including docking, molecular dynamics (MD) simulations and ligand-protein binding energy evaluations, were carried out. NF-κB nuclear translocation and DNA binding activity were reported to be inhibited by different small-molecule ligands. In particular, sesquiterpene lactones, such as helnalin and parthenolide, demonstrated to inhibit NF-κB DNA-binding activity through covalent interactions with the p65 subunit of NF-κB at the level of C38 [68]. The most representative example among NF-κB covalent ligands is probably represented by dehydroxymethylepoxyquinomicin (DHMEQ). In fact, this compound proved to inhibit not only NF-κB DNA-binding activity but also NF-κB nuclear translocation in a dose dependent manner, which was again due to the alkylation of C38 of the p65 subunit [69]. The crucial role of this residue in the NF-κB inhibitory activity of these alkylating agents was demonstrated by mutagenesis studies, which also highlighted the importance for NF-κB DNA-binding activity of C120 of p65 subunit, a residue that is placed in close proximity of C38 [68]. Both residues are located into a site in which p65 subunit takes contacts with DNA in the activated DNA-bound NF-κB p50/p65 heterodimer [56]. 

Therefore, the interaction of these ligands with the p65 subunit of NF-κB should prevent interactions required for the nuclear translocation of the protein and for the correct binding of the heterodimer to DNA. Based on these considerations, it is reasonable to hypothesize that even compounds binding to the p65 subunit through non-covalent interactions in the region where C38 and C120 are located may exert similar inhibition of NF-κB nuclear translocation and DNA-binding activity. 

With the aim of evaluating this hypothesis for OC, we docked the compound into the X-ray structure of the human NF-κB p50/p65 heterodimer [56] in order to evaluate its potential interaction with the DNA-binding domain of NF-κB. To this aim, we applied a validated AUTODOCK protocol for pose prediction analyses, which produced 200 different docking solutions. The generated docking poses were then clustered based on their reciprocal root-mean square (RMSD) deviation, thus obtaining five different clusters of docking solutions (see Materials and Methods for details). For each cluster, the docking pose associated to the highest binding energy was chosen as a representative potential binding mode of OC in complex with p50/p65 heterodimer. In three of the predicted binding modes (clusters 1, 2 and 4), OC was located in the region adjacent to C38 and C120 of the p65 subunit, while in the other two binding modes the ligand was placed into different sites of p65 (cluster 3) and p50 (cluster 5) subunits (Figure S1). With the purpose of analyzing the reliability of the predicted binding modes, the five corresponding ligand-protein complexes (complex 1–5) were studied through a 30 ns MD simulation protocol, as previously performed (see Materials and Methods for details). 

**Figure 9:**
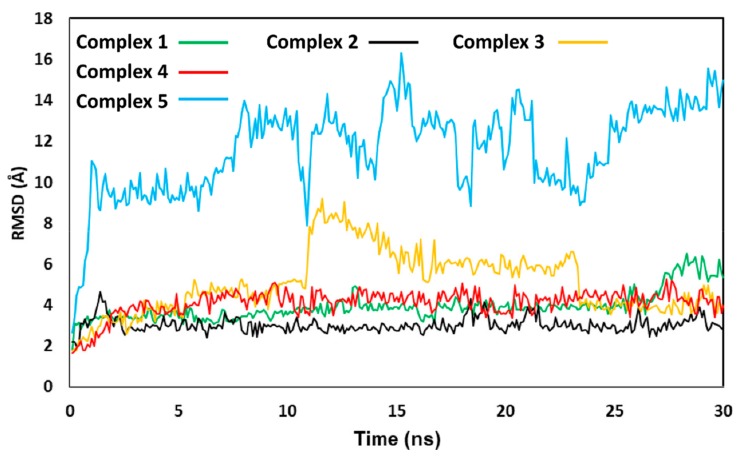
Molecular dynamics (MD) analysis of the 5 OC-p50/p65 complexes. The reciprocal root-mean square deviation (RMSD) of the ligand disposition during the simulation, with respect to the initial docking pose, is shown for the complexes studied.

Results were first analyzed in terms of stability of the ligand binding modes, evaluated based on the RMSD of the disposition of the ligand into the corresponding protein binding site during the simulation, with respect to the starting coordinates. As shown in Figure 9, in all the five different OC-p50/p65 complexes the binding disposition of the ligand underwent at least some adjustment during the simulation, due to the flexibility of the system. However, in complexes 1, 2 and 4, where OC was located in the proximity of C38 and C120 of the p65 subunit, the overall ligand binding mode predicted by docking was maintained. In particular, the binding mode taken on by OC in complex 2 showed to be the most stable, with an average RMSD of the ligand disposition during the MD around 3.0 Å. On the contrary, the RMSD of the ligand coordinates in complex 3 reached values around 6–8 Å for more than 10 ns of simulation, indicating that, in that time lapse, the disposition of OC significantly diverged from the original binding mode predicted by docking. Finally, complex 5 was found to be extremely unstable, since after few ns of simulation the ligand moved about 10–16 Å from its starting disposition. In fact, in this case OC completely lost its interactions with the protein and was released into the bulk solvent. For this reason, this binding mode was considered particularly unreliable, and complex 5 was not taken into account for further analyses.

**Figure 10:**
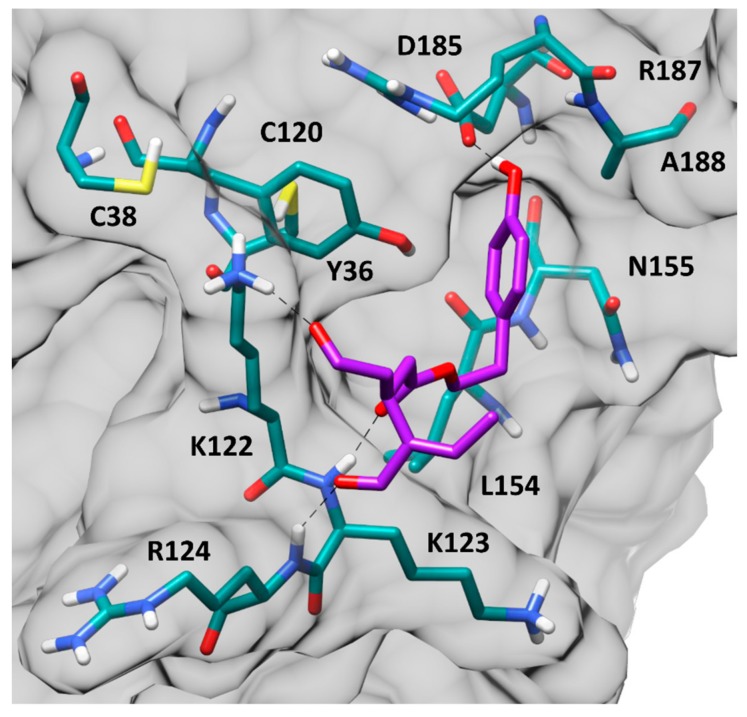
Minimized average structure of OC bound to p65 monomer of the NF-κB p50/p65 heterodimer, derived from the last 20 ns of MD simulation. Hydrogen bonds are represented as black dashed lines, while the protein surface is shown in grey.

The four remaining binding modes, corresponding to complexes 1–4, were analyzed from an energetic point of view, using the Molecular Mechanic-Poisson Boltzmann surface area (MM-PBSA) method, to evaluate their corresponding ligand-protein binding affinities, which were calculated based on the last 20 ns of MD simulation. Binding energy evaluations suggested complex 2 as the most reliable one, since the predicted ligand-protein interaction energy (ΔPBSA = −19.9 kcal/mol) exceeded the binding affinities estimated for the other binding modes of about 3–7 kcal/mol (Table S3). The results of these analyses suggested that OC would most likely interact with the p65 subunit of NF-κB heterodimer, binding to a pocket placed in proximity of C38 and C120. Figure 10 shows the minimized average structure of the human NF-κB p50/p65 heterodimer in complex with OC in the predicted binding mode.

The ligand is placed in a small and shallow pocket of the p65 subunit, located in the proximity of C38 and C120. Three H-bonds anchor the ligand to the protein surface. Precisely, the phenolic hydroxyl group of OC forms a strong H-bond with the carboxylic moiety of D185, which is maintained for the whole MD simulation, while two other very stable H-bonds are established with the backbone nitrogen of K123 and R124 through the ester carbonyl and terminal carbonyl group of the ligand, respectively. In addition, a further H-bond interaction is observed between the positively charged side chain of K122 and the central carbonyl group of OC. This is consistent with the dominant contribute of the electrostatic interactions to the total binding affinity calculated for this ligand-protein complex with the MM-PBSA method (Table S3). Moreover, the portion of the ligand including the ester group and the phenolic ring form hydrophobic interactions with the surrounding residues. In particular, the phenolic ring of OC is positioned inside a small sub-pocket delimited by Y36, C120, N155, D185, R187 and A188, taking van der Waals contacts with these residues. The shape complementarity between the ligand aromatic moiety and the protein sub-pocket positively contributes to the stability of the ligand-protein complex during the MD. Finally, the central chain of the ligand forms lipophilic interactions with Y36, C120, L154 and the side chains of K122 and K123.

In order to explore other possible mechanisms that could contribute to the inhibitory effect of OC and OA on NF-κB activity in human adipocytes, we evaluated the potential interaction of OC with IKKβ, which regulates NF-κB activity. By employing the same docking procedure used to study OC-p50/p65 binding interaction, OC was docked into the active site of IKKβ and the best predicted docking pose was then subjected to the same 30 ns MD simulation protocol applied on OC-p50/p65 complexes. This analysis suggested that OC would be able to form interactions with key residues in the catalytic site of the protein, thus showing the potential to behave as an IKKβ inhibitor. In particular, OC was predicted to form an H-bond interaction with C99, belonging to the hinge region of the protein, and two additional H-bonds with E61 and the catalytic lysine K44 (Figure S2). An inhibitory effect of OC on IKKβ activity would potentiate NF-κB inhibition limiting the release of NF-κB from its cytoplasmic inactivated complex with IκB proteins and its translocation to the cell nucleus.

## 4. Discussion

This study shows that OC and OA, two secoiridoid polyphenols typically present in EVOO, a basic component of the Mediterranean diet, present anti-inflammatory effects in human adipocytes challenged with TNF-α, a prototypic inflammatory stimulus. TNF-α is overexpressed in AT during obesity, and is causally linked to AT inflammation [70]. We showed that OC and OA: (1) attenuated TNF-α-induced expression of genes and miRNAs involved in AT inflammation and related adipocyte dysfunction; (2) reduced the activation of NF-κB pathway; and (3) may prevent NF-κB activity by directly interacting with the p65 subunit. These newly discovered effects of OC and OA expand the potential health-promoting properties of these compounds, and could contribute to explaining the cardiometabolic benefit of EVOO consumption. 

A dysregulated pattern of adipocyte gene expression is involved in the initiation and progression of AT inflammation associated with obesity and its metabolic and vascular complications. During the course of obesity, AT is characterized by adipocyte hypertrophy, oxidative stress, and subsequent increased angiogenesis, immune cell infiltration, and tissue remodeling. Mechanistically, adipocyte hypertrophy is accompanied by the upregulation of proinflammatory cytokines and chemokines, such as TNF-α, IL-1, IL-6, MCP-1, CXCL-10, M-CSF, angiogenic factors such as VEGF, matrix-degrading and proinflammatory enzymes such as MMPs and COX-2, respectively, and pro-oxidant enzymes, including NADPH oxidase. Conversely, adipocyte hypertrophy is also accompanied by an opposite downregulation of anti-inflammatory and insulin-sensitizing adipokines, such as adiponectin, and impaired antioxidant defense. The increased oxidative stress and altered pattern of secretory products by adipocytes participate in the establishment of an autocrine/paracrine loop, with further adipocyte and macrophage pro-inflammatory activation and derangement, as well as of an “endocrine” signaling, propagating AT dysfunction to distant metabolic and vascular organs [40].

Increased production of cytokines and chemokines in obese AT has been implicated in the regulation of monocyte recruitment to the AT and pro-inflammatory activation, that further exacerbates AT inflammation and insulin resistance [71,72]. In particular, the obligate role in AT macrophage recruitment and inflammation of MCP-1 [73,74], a potent chemoattractant for monocytes/macrophages, as well as M-CSF, a critical regulator of macrophage development and survival [72], has been demonstrated in obesity in animal models. Furthermore, overproduction of MCP-1 by AT may also exert an endocrine role via the systemic circulation, inducing insulin resistance in the skeletal muscle and the liver [74,75], and also negatively impacting on the vascular wall, eventually relating obesity and related cardiovascular diseases [76]. Other chemokines, including CXCL-10 [77], play a role in orchestrating monocyte/macrophage chemotaxis. 

We here show that OC and OA almost totally prevented the mRNA upregulation of IL-1β, MCP-1, CXCL-10 and, for OC only, M-CSF as well as MCP-1 protein secretion in inflamed adipocytes, thus highlighting the potential for these compounds to blunt adipocyte dysfunction and the associated pro-inflammatory cascade occurring during obesity. 

A similar anti-inflammatory effect on gene expression of cytokines, chemokines and other pro-inflammatory markers has been previously found for both compounds in different cell models. OC was able to inhibit gene expression of IL-6, MIP-1α in murine chondrocytes and macrophages challenged with lipopolysaccharide (LPS), and IL-1β, IL-6, TNF-α, GM-CSF, IL-8, CCL3, LCN2, MMP-13, ADAMTS-5 in LPS-stimulated macrophages [78,79]. OA has been shown to inhibit TNF-α- and lipopolysaccharide (LPS)-induced MCP-1 and adhesion molecules (ICAM-1, VCAM-1, and E-selectin) mRNA expression in endothelial cells and consequent monocyte adhesion to the endothelium [36]. It also prevented the expression of the adhesion molecule CD11b/CD18, and the release of elastase, MMP-9, and IL-8 in human neutrophils [80], and enhanced the anti-inflammatory potential of human macrophages, by upregulating the expression of CD163, as well as IL-10 and heme oxygenase (HO)-1 secretion [81], with a potential atherosclerotic plaque-stabilizing effect [82]. 

In accordance with the early demonstration of an anti-inflammatory action of OC and OA by inhibiting the activity of the pro-inflammatory COX [33] and 5-lipoxygenase (LOX) [83] enzymes in cell-free enzymatic assays, as well as the expression of COX-2 in LPS-challenged human monocytes (with a 96% and 88% inhibition for OC and OA, respectively) [84] and chondrocytes [79], we here show that OC and OA were able to abolish COX-2 mRNA upregulation induced by TNF-α in human SGBS adipocytes. This effect could have therapeutic implications for obesity-related inflammation because COX-2-derived prostanoids, mainly prostaglandin E_2_ (PGE_2_), play a significant role in mediating macrophage infiltration and inflammation in the visceral fat and the development of AT and systemic insulin resistance and fatty liver [85,86]. From a mechanistic point of view, using specific COX-2 and PGE_2_/EP3 receptor signaling inhibitors, the adipocyte COX-2/PGE_2_/EP3 receptor-mediated pathway during hypertrophy and hypoxia was involved in the activation of NF-κB and hypoxia-inducible factor (HIF)-1α, with consequent increased production of MCP-1, TNF-α, leptin and reactive oxygen species (ROS), as well as decreased adiponectin expression [86]. Accordingly, the COX-2 and EP3 mRNA levels were positively correlated with the body mass index (BMI) in human subjects, corroborating animal and in vitro results [86]. Moreover, COX-2-derived prostanoids have been implicated in MMP induction in vascular and inflammatory cells, thus promoting angiogenesis and tissue remodeling [31]. 

Fat accumulation is a complex process involving adipogenesis, angiogenesis and proteolytic remodeling of the extracellular matrix, where a critical role is exerted by adipocyte expression and release of MMPs, including the gelatinase MMP-2 [87], as well as the angiogenic growth factor VEGF [88]. In vitro and in vivo studies suggest that MMP-2 may be a key regulator of adipocyte differentiation and AT expansion, through the degradation of the extracellular matrix and basement membrane components, the activation of latent growth factors, the stimulation of angiogenesis and adipogenesis [87,89]. Moreover, VEGF expression in AT, inducible by inflammatory cytokines in both the stromal-vascular fraction and mature adipocytes, could contribute to the vascularization preceding AT growth [88]. Although metabolic consequences of manipulating VEGF expression in mice are not consistent in the literature, a recent study has found that adipose-specific VEGF repression in mice leads to white AT browning, uncoupling protein(UCP)-1 upregulation accompanied by reduced lipid accumulation in AT, resistance to obesity, and increased insulin sensitivity. VEGF-A, the isoform here measured, binds both the tyrosine kinase receptors KDR (VEGF-R2) and VEGF-R1, expressed mainly on the vascular endothelium. However, only blockade of KDR, and not VEGF-R1, restricts AT expansion and limits diet-induced fat tissue expansion. Although a previous study failed to show KDR expression in murine pre-adipocytes and ruled out an autocrine stimulation of VEGF on adipocyte function, we could detect the mRNA expression of KDR as inducible by TNF-α in human SGBS adipocytes, but the potential downstream effect(s) of VEGF/KDR signaling activation in adipocytes is (are) unknown and should be further investigated. Relevant to these issues, we here demonstrated that OC and OA also strongly attenuated the TNF-α-induced upregulation of MMP-2 as well as VEGF and KDR mRNA levels in human adipocytes. A similar modulatory effect on MMP-2 and VEGF expression has been recently reported for OC in melanoma cells, via the suppression of STAT3 transcriptional pathway.

Obesity development is associated with unbalanced overproduction of ROS [90], which are implicated in the development of adipocyte inflammation, recruitment of macrophages to AT, and whole-body insulin resistance, [91,92]. In particular, a role for NOX, a membrane-bound enzyme complex that produces superoxide by transferring electrons from NAD(P)H to molecular oxygen, has recently emerged [92]. NOX-4 is the major NOX isoform in cultured murine and human adipocytes, and NOX-4-derived ROS mediate the increased expression of chemotactic factors in cultured adipocytes exposed to nutrient excess [93]. Diet-induced obesity in mice is accompanied by increased AT NOX-4 activity, the inhibition of which leads to decreased ROS and AT-inflammation [91]. A role for the NOX-2 isoform of NADPH oxidase has also been inferred, as deficiency of NOX-2 attenuated AT inflammation and insulin resistance in mice fed a high-fat diet [94]. We here provide the first demonstration of the suppression by OC and OA of the TNF-α-induced upregulation of both NOX-2 and, more strongly, NOX-4 mRNA in human adipocytes. This previously unappreciated effect on pro-oxidant gene expression could complement the antioxidant properties demonstrated for both compounds [95,96]. Indeed, OC and OA have been found to inhibit NADPH oxidase activity and intracellular superoxide anion production in human monocytes [84]. This occurred at a relatively high concentration (100 µmol/L) that, in the case of OC, partially reduced cell viability [84], at variance from our experimental condition in which the reduced expression of NOX-4 by 25 µmol/L OC or OA was not associated with any effect on adipocyte viability. Furthermore, OA was seen to prevent H_2_O_2_-induced DNA damage in monocytes [97], and reduced ROS production and myeloperoxidase release by human neutrophils [80]. OA was also found to prevent angiotensin II-induced ROS production and senescence in endothelial progenitor cells via the activation of the antioxidant transcription factor nuclear factor-E2-related factor 2 (Nrf2) and the increased expression of HO-1 [98]. These antioxidant effects of OC and OA are concordant with the ability of polyphenol extracts from EVOO to inhibit the activity of NADPH oxidase and the expression of NOX-4 and NOX-2 in endothelial cells [99]. Concordantly, EVOO was able to counteract postprandial oxidative stress by reducing NOX-2 activity in healthy subjects [100]. In agreement with a similar previously reported effect by the EVOO simple polyphenol hydroxytyrosol [31], we here also show that OC and OA prevented the TNF-α-induced upregulation of antioxidant enzymes, such as GPX and mitochondrial SOD, which play a role in detoxifying superoxide anion and hydrogen peroxide (and organic hydroperoxides), respectively. This modulatory effect suggests the attenuation of cell oxidative stress and a restoration of the basal antioxidant defense in the presence of anti-inflammatory and antioxidant polyphenols.

Although not directly tested in the present study, the modulation of gene expression associated with processes involved in adipocyte dysfunction and inflammation could lead to an improved adipocyte secretory profile, reduced production of ROS, improved metabolic function, as well as decreased chemotaxis and activation of macrophages. Collectively, these data may lay down the basic foundations for further animal and human studies on the cardiometabolic benefit of OC and OA as either isolated compounds or as food ingredients.

To gain further molecular insight into the anti-inflammatory activity of OC and OA, we studied the effect of these polyphenols on the activation of NF-κB, which transcriptionally regulates a plethora of pro-inflammatory genes including those here assessed, and functions as a link between inflammation and dysmetabolism in obesity [6,101]. The NF-κB family is a heterogeneous set of transcription factors that dimerize in different combinations at a common dimerization domain called Rel homology domain [102]. Five mammalian NF-κB proteins have been identified, including p65 (also named as RelA), RelB, c-Rel, p50, and p52, which share a conserved DNA-binding domain. With the exception of p52, these proteins are constitutively present in the cytoplasm, bound to inhibitory proteins, i.e., inhibitor of κB (IκB)-α and IκB-β. Activation of the NF-κB pathway by most stimuli, including TNF-α, involves ROS-sensitive pathways and leads to IκB-α and IκB-β degradation and the consequent translocation of p50/p65 heterodimers into the nuclei, where they bind to promoter regions of genes encoding for effectors of the inflammatory response, such as cytokines, chemokines, adhesion molecules, acute-phase proteins, inducible enzymes (COX-2, inducible nitric oxide synthase, etc.), and proangiogenic growth factors [5]. NF-κB is an attractive pharmacological and nutritional target in the prevention and treatment of inflammatory diseases, and its inhibition by dietary polyphenols has emerged as a common denominator of most of the anti-inflammatory properties of these compounds [103]. 

Previous studies have reported the suppression of the NF-κB pathway by hydroxytyrosol and oleuropein in several cell models [31], and by OC in LPS-challenged human chondrocytes [104,105]. Concordantly, we here found, for the first time, that OC and OA are able to inhibit NF-κB activation in TNF-α-stimulated human adipocytes, as demonstrated by reduced DNA binding activity of the p65 subunit from OC- and OA-treated nuclear extracts. This may represent a mechanism underlying the general downregulation by these compounds of genes involved in AT inflammation. The interference with NF-κB activation may occur through (a) direct targeting of the DNA binding activity of individual NF-κB proteins; (b) blocking the nuclear translocation of NF-κB by inhibiting the nuclear import system; (c) stabilizing IκBα protein; (d) targeting cytosolic signaling nodes upstream of NF-κB activation, such as IκB kinase (IKK)β [102]. We cannot rule out the involvement of oxidative stress inhibition by OC and OA in the observed attenuation of NF-κB activation. Moreover, polyphenols might also act by directly interacting with protein targets to inhibit their function [106]. Previous studies demonstrated that small-molecule ligands can inhibit both the DNA binding activity and nuclear translocation of NF-κB by interacting with a specific region of the p65 subunit encompassing the cysteine residues C38 and C120. Therefore, we applied a molecular modelling protocol including docking studies, MD simulations and binding energy evaluations with the aim of investigating the reliability of a direct interaction between EVOO polyphenols and NF-κB, which could thus represent a potential molecular mechanism at the basis of the activity of these compounds. For these studies, we focused on OC, which is a prototypical secoiridoid structurally similar to—but more active than—OA. Our computational protocol, which evaluated multiple potential binding modes of OC in complex with the human NF-κB p50/p65 heterodimer, suggested that the compound would most likely interact with the p65 subunit of NF-κB, binding to a pocket located in the proximity of C38 and C120, as reported for known NF-κB inhibitors. Moreover, additional computational studies suggested that OC could favorably interact with the catalytic site of IKKβ and act as an IKKβ inhibitor, thus hampering the phosphorylation of IκB proteins and the release of activated NF-κB ready to be translocated to the cell nucleus. Although not formally tested in the present study, we could infer similar NF-κB binding modes by OA. The results of our molecular modeling studies suggest novel possible modes of action at the basis of the anti-inflammatory protective effect of EVOO polyphenols. Further studies will be aimed at addressing a more in-depth evaluation of these as well as other possible mechanisms of action of OC, OA and other EVOO polyphenols. 

Although improvements in AT inflammation in obesity can translate into beneficial metabolic effects, cellular and animal studies suggest that dietary polyphenols may be effective in contrasting obesity and its metabolic derangements by directly modulating energy metabolism, lipid/lipoprotein metabolism, glucose homeostasis, and insulin sensitivity, in relevant tissues including the AT and the liver [107]. We therefore examined the effect of OC and OA on the gene expression of a metabolic effector, i.e., PPARγ, primarily expressed in the AT and intertwined with adipogenesis and insulin sensitivity. We found that OC and OA counteracted the inhibition of PPARγ expression in response to TNF-α. PPARγ, a member of the PPAR nuclear receptor subfamily, is a transcription factor controlling the expression of genes involved in lipid metabolism, glucose homeostasis, insulin signaling, and cellular differentiation [108]. PPARγ agonists, such as rosiglitazone and pioglitazone, are therapeutic agents for type 2 diabetes [108]. Besides energy metabolism, another well-established function of PPARγ is the inhibition of inflammatory gene expression through the interference with specific pro-inflammatory transcription factors including NF-κB, by inducing its negative regulator IκBα or interacting with NF-κB and its regulatory proteins leading to NF-κB inactivation (i.e., trans-repression) [109]. Therefore, PPARγ improvement by OC and OA offers an additional mechanism for their anti-inflammatory action and the inhibition of NF-κB signaling. A similar improvement of PPARγ expression has been previously reported for OC-rich EVOO in the brain of Alzheimer’s disease mice model [110], and for the EVOO simple phenol hydroxytyrosol, a precursor of OA, in TNF-α-stimulated SGBS cells [45]. A very recent study documented an OA-mediated down-regulation of PPARγ in murine differentiating 3T3-L1 pre-adipocytes and in vivo in high-fat diet-fed mice, which was accompanied by reduced adipogenesis and weight gain and improved insulin resistance [111]. Here we provide the first demonstration of PPARγ upregulation by OC and OA in human mature adipocytes under inflammatory condition, with implication for attenuating inflammation and adipocyte dysfunction. In order to further evaluate OC and OA role in obesity and dysmetabolism, our following studies will be aimed at assessing the effects of OA and OC on other major molecular regulators of metabolic functions, including adipogenesis (e.g., C/EBPα, fatty acid binding protein), lipogenesis (e.g., SREBP1c, fatty acid synthase [FAS], acetyl-CoA carboxylase [ACC], etc.), lipolysis (e.g., adipose triglyceride lipase [ATGL] and hormone sensitive lipase [HSL]), insulin signaling and glucose uptake/metabolism (e.g., IRS-1/2, GLUT-4), as well as fatty acid oxidation and energy expenditure (e.g., PPARα, 5′ adenosine monophosphate-activated protein kinase [AMPK], PPARγ coativator [PGC]-1α, uncoupling protein-1/2). 

Another notable observation of the present study is the involvement of miRNAs in the molecular anti-inflammatory response observed in adipocytes following OC and OC treatment. Currently, miRNAs have been found to be regulated by some natural compounds. By altering the expression of miRNAs and influencing the downstream signaling pathways or target genes, several natural compounds exhibit their bioactivity in the prevention, diagnosis, therapy, prognosis and drug resistance of human diseases [112]. The here-tested EVOO polyphenols demonstrated the ability to modulate miRNAs linked to inflammation, i.e., miR-34a, miR-155 and let-7c. Indeed, the expression of these miRNAs is significantly modulated by TNF-α in human adipocytes toward a pro-inflammatory mode, and is closely associated with NF-κB signaling. 

Emerging evidence suggests that miR-34a levels are elevated by NF-κB through directly binding to its promoter [26]. Furthermore, miR-34a directly interacts with sirtuin 1 (Sirt1), which leads to the rapid suppression of NF-κB [29]. Therefore, it is conceivable the activation of a biological autocrine loop, whereby NF-κB increases miR-34a levels and miR-34a induces the activation of NF-κB. 

Further, miR-155 is starkly connected to the inflammatory process and the NF-κB pathway [25,27,28]. A recent article reported that NF-κB-p65 interacts with the promoter site of miR-155, so that NF-κB activation increases levels of miR-155. Moreover, miR-155 may regulate the expression of IκB kinase (IKKβ and IKKε), which leads to the repression or, at least, the limitation of NF-κB activation) [27]. miR-155 has also been seen to repress B-cell leukemia/lymphoma(BCL) 6, a negative regulator of NF-κB signaling, thus promoting inflammatory pathways in macrophages [113]. Taken together, these data indicate the existence of a NF-κB/miR-155 pathway [114]. In our model of TNF-α-stimulated adipocytes, the phenolic compounds OC and OA showed the ability to counteract the increased expression of miR-34a and miR-155, potentially interrupting the pro-inflammatory NF-κB/miR-34a/miR-155 loop. Furthermore, PPARγ is a recognized direct target of both miR-155 [19] and miR-34a [115]. Therefore, the downregulation of mir-155 and mir-34a by OC and OA could mediate, at least in part, the here observed recovery of PPARγ expression by both compounds.

Another miRNA tightly connected with the NF-κB pathway is let-7 [24]. Indeed, NF-κB directly activates Lin28B, a strong inhibitor of let-7 expression at the transcriptional and post-transcriptional levels [24,116]. The attenuated NF-κB signaling induced by OC and OA led to increased let-7c levels, thus interfering with the feedback loop. Moreover, increased levels of let7-c are in line with the decreased levels of its direct target, COX-2 (miRTarBase ID: MIRT051845), confirming the anti-inflammation potential of let-7c modulation by OC and OA.

Notably, adipocyte-derived miRNAs and exosome-derived miRNAs isolated from conditioned media showed an overlapping expression pattern. Indeed, miRNAs are also present in the extracellular environment in membrane-covered microvesicles as exosomes [117]. Recent studies have revealed that microvesicles containing miRNAs can be secreted by adipocytes and act as endocrine and/or paracrine signaling molecules inside recipient cells [12]. By this way, miRNAs deliver effects at a distance after being vehicled in body fluids [118]. Therefore, the beneficial modulation by OC and OA of exosomal miR-34a, miR-155 and let-7c suggests the ability for these compounds also to control cell-to-cell communication.

The bioinformatic analysis of biological pathways predicted to be associated with the regulation of miR-34a, miR-155 and let-7c confirms the involvement of pathways belonging to the inflammatory process, including, regarding miR-155 and miR-34a: cytokine-mediated signaling, response to TNF-α pathway, angiogenesis and vascular remodeling, and positive regulation of chemotaxis and NF-κB activity; and regarding let7-c: cell communication, regulation of cell cycle and response to cytokines, and extracellular matrix organization. Since these processes are also profoundly implicated in the AT pathophysiology during obesity development, through the regulation of miR-34a, miR-155 and let-7c, OC and OA could exert an overall blocking effect of inflammatory and dysmetabolic pathways in human adipocytes. Therefore, the miRNAs here investigated may cooperate in the regulation of the observed anti-inflammatory effects of OC and OA in human adipocytes.

An important limitation of the present study is the use of OC and OA parent compounds that may not be the major bioavailable forms in vivo. OC and OA—the latter also deriving from oleuropein degradation—are stable in the acidic condition of the stomach, reach the small intestine in an unchanged form and enter the systemic circulation mainly as glucuronides [119,120]. Of note, the inflammatory cells may secrete lysosomal β-glucuronidase into the extracellular space [121], thus potentially increasing the local concentration of the free forms of polyphenols. A recent study reports that OA is readily absorbed and metabolized to hydroxytyrosol, homovanillic acid, and homovanillyl alcohol after oral administration in rats [122]. However, metabolism and bioavailability of these compounds, mostly OC, need to be firmly established to substantiate their health effects. Although only three miRNAs genes were here investigated, our results provide preliminary evidence that OC and OA could exert anti-inflammatory efficacy targeting miRNAs in adipocytes, thus paving the way for further studies.

**Figure 11:**
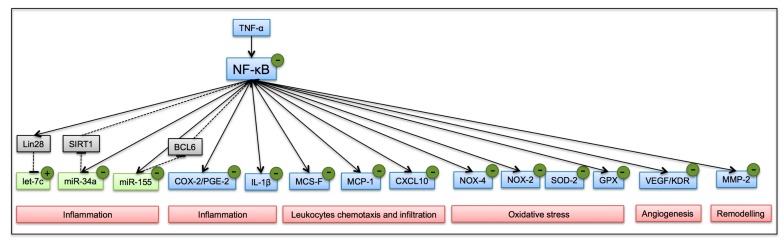
A working model of action for OC and OA in TNF-α-stimulated adipocytes. Continuous line, active; dashed line, inhibit; blue box, gene; green box, miRNA; gray box, intermediate knots; red box, processes; polyphenols effect: (−) decrease; (+) increase.

Overall, our findings outline a working model for OC and OA, demonstrating that polyphenols isolated from EVOO, OC and OA, counteract inflammation by attenuating NF-κB activation, the expression of inflammatory cell and exosomal miRNAs, and concomitantly modulate the expression of pro-inflammatory genes toward a protective profile in inflamed adipocytes (Figure 11). These results provide evidence of unappreciated protective effects and active ingredients mediating the cardio-metabolic benefits of EVOO consumption, suggesting OC and OA as novel dietary tools to limit metabolic inflammation associated with obesity.

## Supplementary Materials

The following are available online at https://www.mdpi.com/2072-6643/11/12/2855/s1, Figure S1: Oleocanthal (OC)-p50/p56 complexes analyzed with Molecular dynamics (MD). The five OC-p50/p65 complexes studied with MD simulations are shown, together with the DNA fragment bound to the heterodimer in the reference X-ray structure. In complex 1 (A), complex 2 (B), complex 3 (C), complex 4 (D) and complex 5 (E), the ligand is shown as spheres and colored green, black, orange, red and blue, respectively, while the bound DNA fragment (F) is colored in dark cyan. In all complexes, the p50 and p65 subunits are shown as pink and purple ribbons respectively, while cys38 and cys120 are shown as yellow spheres, Figure S2: Minimized average structure of OC bound to IKKβ catalytic site, derived from the last 20 ns of MD simulation. Hydrogen bonds are represented as black dashed lines. Table S1: Gene ontology (GO) Biological Processes (BP) significantly associated to miR-155-5p and miR-34a-5p, Table S2: Gene ontology (GO) Biological Processes (BP) significantly associated to let-7c-5p, Table S3: Molecular Mechanic-Poisson Boltzmann surface area (MM-PBSA) evaluation. MM-PBSA results for four analyzed oleocanthal (OC)-p50/p65 complexes. ΔPBSA is the sum of the electrostatic (ELE), van der Waals (VDW), polar (EPB) and non-polar (ENPOLAR) solvation free energy. Data are expressed as kcal·mol^−1^.
