# Supplementary material for: The Extra-Virgin Olive Oil Polyphenols Oleocanthal and Oleacein Counteract Inflammation-Related Gene and miRNA Expression in Adipocytes by Attenuating NF-κB Activation
Source: Nutrients. 2019 Nov 21;11(12):2855. doi: 10.3390/nu11122855 (PMC6950227; doi:10.3390/nu11122855)
Supplement: Supplementary file 1 [file nutrients-11-02855-s001.pdf]

Supporting Information

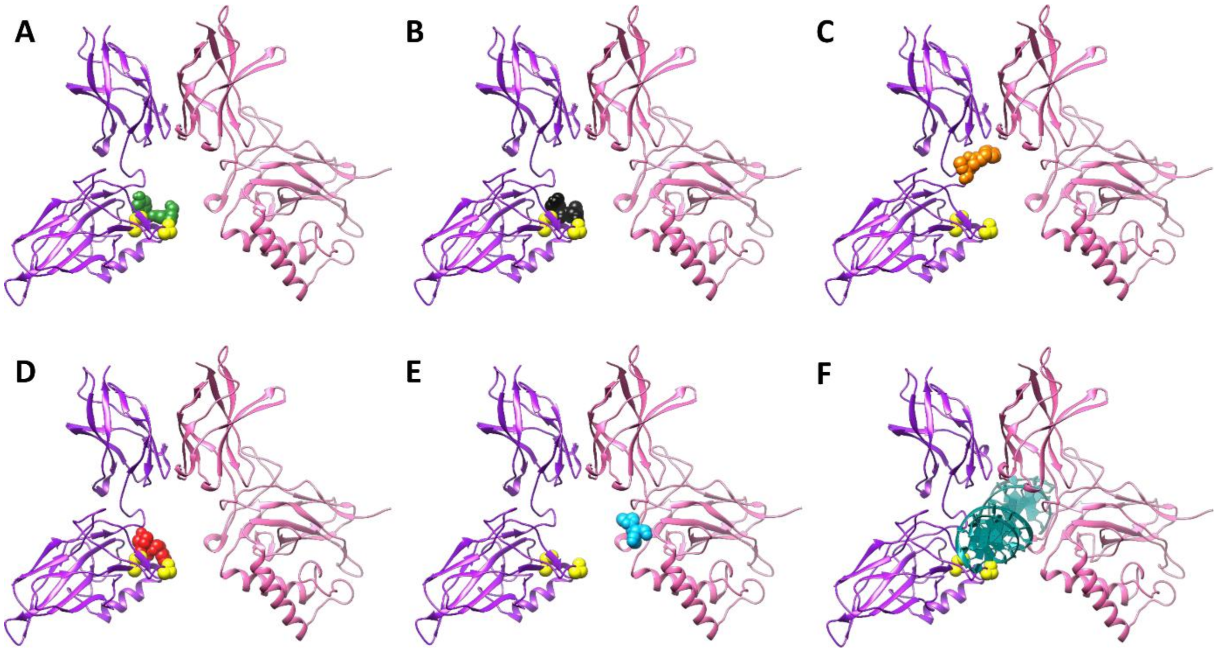

Figure S1. Oleocanthal (OC)-p50/p56 complexes analyzed with Molecular dynamics (MD). The five OC-p50/p65 complexes studied with MD simulations are shown, together with the DNA fragment bound to the heterodimer in the reference X-ray structure. In complex 1 (**A**), complex 2 (**B**), complex 3 (**C**), complex 4 (**D**) and complex 5 (**E**), the ligand is shown as spheres and colored green, black, orange, red and blue, respectively, while the bound DNA fragment (**F**) is colored in dark cyan. In all complexes, the p50 and p65 subunits are shown as pink and purple ribbons respectively, while cys38 and cys120 are shown as yellow spheres.

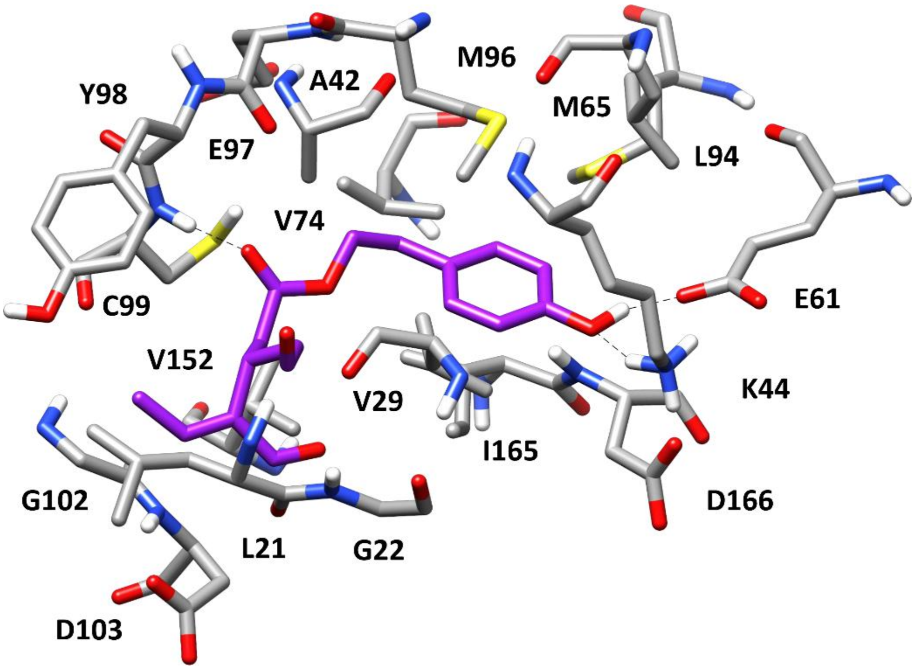

Figure S2. Minimized average structure of OC bound to IKKβ catalytic site, derived from the last 20 ns of MD simulation. Hydrogen bonds are represented as black dashed lines.

Table S1. Gene ontology (GO) of Biological Processes (BP) significantly associated to miR-155-5p and miR-34a-5p.

Current Gene List: miR155-5p + miR34a-5p
Current Background: Homo sapiens

Term	Count	P-Value	Fold Enrichment	Benjamini	FDR
positive regulation of transcription from RNA polymerase II promoter	142	0,0000	2,2	2,70E-16	1,30E-16
positive regulation of transcription, DNA-templated	85	0,0000	2,5	7,20E-12	6,80E-12
transcription from RNA polymerase II promoter	75	0,0000	2,2	1,00E-07	1,40E-07
negative regulation of transcription from RNA polymerase II promoter	92	0,0000	2	6,60E-07	1,20E-06
negative regulation of transcription, DNA-templated	68	0,0000	2,1	9,70E-06	2,30E-05
regulation of transcription from RNA polymerase II promoter	57	0,0000	2	8,10E-04	2,30E-03
transcription, DNA-templated	174	0,0000	1,4	7,20E-03	2,40E-02
neural crest cell migration	13	0,0000	4,4	1,10E-02	4,00E-02
BMP signaling pathway	17	0,0000	3,4	1,10E-02	4,70E-02
Ras protein signal transduction	16	0,0000	3,5	1,40E-02	6,70E-02
intracellular signal transduction	49	0,0000	1,9	1,30E-02	7,00E-02
positive regulation of mammary gland epithelial cell proliferation	6	0,0001	11,5	1,80E-02	1,00E-01
protein phosphorylation	53	0,0001	1,8	1,80E-02	1,10E-01
positive regulation of osteoblast differentiation	14	0,0001	3,6	2,90E-02	1,90E-01
neuron migration	19	0,0001	2,8	3,70E-02	2,60E-01
SMAD protein signal transduction	14	0,0002	3,5	3,60E-02	2,80E-01
Wnt signaling pathway	27	0,0002	2,2	4,70E-02	3,80E-01
epidermal growth factor receptor signaling pathway	13	0,0002	3,6	4,60E-02	4,00E-01
signal transduction	107	0,0002	1,4	4,60E-02	4,20E-01
steroid hormone mediated signaling pathway	13	0,0003	3,5	4,90E-02	4,70E-01
canonical Wnt signaling pathway	16	0,0003	3	5,00E-02	5,10E-01
small GTPase mediated signal transduction	32	0,0003	2	5,70E-02	6,00E-01
protein ubiquitination	42	0,0003	1,8	5,50E-02	6,20E-01
transport	41	0,0003	1,8	5,50E-02	6,40E-01
activin receptor signaling pathway	7	0,0003	6,7	5,30E-02	6,40E-01
positive regulation of Wnt signaling pathway	10	0,0004	4,3	5,80E-02	7,20E-01
peptidyl-serine phosphorylation	20	0,0005	2,5	6,60E-02	8,60E-01
negative regulation of gene expression	21	0,0006	2,3	7,70E-02	1,10E+00
intracellular receptor signaling pathway	10	0,0006	4	7,90E-02	1,10E+00
establishment of cell polarity	8	0,0006	5,1	8,00E-02	1,20E+00
axon extension	8	0,0006	5,1	8,00E-02	1,20E+00
axon guidance	23	0,0007	2,2	8,00E-02	1,20E+00
negative regulation of cell proliferation	44	0,0007	1,7	8,20E-02	1,30E+00
transforming growth factor beta receptor signaling pathway	16	0,0009	2,7	9,70E-02	1,60E+00
somatic stem cell population maintenance	13	0,0009	3,1	9,90E-02	1,70E+00
Positive regulation of NF-kappaB transcription factor activity 16	25	0,0009	2,1	9,70E-02	1,70E+00
regulation of exocytosis	8	0,0011	4,7	1,10E-01	2,00E+00
protein targeting to plasma membrane	8	0,0011	4,7	1,10E-01	2,00E+00
T cell differentiation in thymus	8	0,0014	4,5	1,40E-01	2,50E+00
embryonic hindlimb morphogenesis	8	0,0017	4,4	1,60E-01	3,20E+00
regulation of transcription, DNA-templated	127	0,0017	1,3	1,60E-01	3,20E+00
chemical synaptic transmission	29	0,0020	1,9	1,80E-01	3,70E+00
nervous system development	33	0,0021	1,8	1,80E-01	3,80E+00
negative regulation of canonical Wnt signaling pathway	22	0,0021	2,1	1,80E-01	3,90E+00
cardiac conduction	10	0,0022	3,4	1,80E-01	3,90E+00
activation of protein kinase activity	10	0,0022	3,4	1,80E-01	3,90E+00
protein transport	42	0,0022	1,6	1,80E-01	4,00E+00
beta-catenin destruction complex assembly	4	0,0025	12,3	2,00E-01	4,50E+00
peptidyl-threonine phosphorylation	9	0,0027	3,6	2,10E-01	4,90E+00
embryonic pattern specification	7	0,0029	4,7	2,20E-01	5,30E+00
palate development	13	0,0036	2,6	2,60E-01	6,40E+00
lung development	13	0,0036	2,6	2,60E-01	6,40E+00
regulation of insulin secretion	12	0,0038	2,7	2,60E-01	6,80E+00
viral process	33	0,0040	1,7	2,70E-01	7,10E+00
heart development	23	0,0040	1,9	2,70E-01	7,20E+00
positive regulation of long-term synaptic potentiation	5	0,0041	7	2,70E-01	7,30E+00
cartilage development	11	0,0045	2,9	2,90E-01	8,00E+00
cell cycle arrest	19	0,0047	2,1	2,90E-01	8,40E+00
positive regulation of GTPase activity	54	0,0048	1,5	2,90E-01	8,50E+00
positive regulation of endothelial cell proliferation	12	0,0048	2,7	2,90E-01	8,50E+00
branching involved in ureteric bud morphogenesis	9	0,0052	3,3	3,00E-01	9,10E+00
in utero embryonic development	23	0,0052	1,9	3,00E-01	9,30E+00
response to hydrogen peroxide	10	0,0052	3	3,00E-01	9,30E+00
adult behavior	7	0,0056	4,1	3,10E-01	1,00E+01
protein homooligomerization	22	0,0057	1,9	3,10E-01	1,00E+01
angiogenesis	26	0,0057	1,8	3,10E-01	1,00E+01
positive regulation of receptor-mediated endocytosis	6	0,0063	4,8	3,30E-01	1,10E+01
positive regulation of Ras protein signal transduction	6	0,0063	4,8	3,30E-01	1,10E+01
positive regulation of TOR signaling	7	0,0069	4	3,50E-01	1,20E+01
signal transduction by protein phosphorylation	9	0,0069	3,1	3,40E-01	1,20E+01
post-embryonic development	12	0,0074	2,5	3,60E-01	1,30E+01
vocal learning	4	0,0079	8,8	3,70E-01	1,40E+01
glial cell proliferation	4	0,0079	8,8	3,70E-01	1,40E+01
retina vasculature development in camera-type eye	4	0,0079	8,8	3,70E-01	1,40E+01
cellular hypotonic response	4	0,0079	8,8	3,70E-01	1,40E+01
positive regulation of pri-miRNA transcription from RNA polymerase II promoter	6	0,0079	4,6	3,70E-01	1,40E+01
membrane depolarization during cardiac muscle cell action potential	5	0,0080	5,9	3,70E-01	1,40E+01
determination of left/right symmetry	10	0,0087	2,8	3,90E-01	1,50E+01
cell proliferation	37	0,0090	1,5	3,90E-01	1,50E+01
regulation of cellular response to heat	12	0,0090	2,5	3,90E-01	1,60E+01
histone deacetylation	9	0,0091	3	3,90E-01	1,60E+01
chromatin remodeling	13	0,0097	2,3	4,10E-01	1,70E+01
cerebral cortex development	10	0,0098	2,7	4,00E-01	1,70E+01
negative regulation of neuron differentiation	10	0,0098	2,7	4,00E-01	1,70E+01
ventricular septum morphogenesis	7	0,0099	3,7	4,00E-01	1,70E+01
neuronal stem cell population maintenance	6	0,0099	4,4	4,00E-01	1,70E+01
ureteric bud development	8	0,0100	3,2	4,10E-01	1,80E+01
vocalization behavior	5	0,0110	5,5	4,10E-01	1,80E+01
TOR signaling	5	0,0110	5,5	4,10E-01	1,80E+01
calcium ion transport into cytosol	5	0,0110	5,5	4,10E-01	1,80E+01
learning	10	0,0110	2,7	4,20E-01	1,80E+01
embryonic skeletal system development	7	0,0120	3,6	4,40E-01	2,00E+01
positive regulation of neuron differentiation	12	0,0120	2,4	4,40E-01	2,00E+01
regulation of phosphatidylinositol 3-kinase signaling	12	0,0120	2,4	4,40E-01	2,00E+01
response to progesterone	8	0,0120	3,1	4,40E-01	2,00E+01
gamma-aminobutyric acid signaling pathway	6	0,0120	4,2	4,40E-01	2,00E+01
beta-catenin destruction complex disassembly	6	0,0120	4,2	4,40E-01	2,00E+01
calcium ion transmembrane transport via high voltage-gated calcium channel	3	0,0120	15,3	4,30E-01	2,00E+01
response to glucose	11	0,0120	2,5	4,30E-01	2,10E+01
MAPK cascade	28	0,0120	1,6	4,30E-01	2,10E+01

brain development	22	0,0120	1,8	4,30E-01	2,10E+01
embryonic cranial skeleton morphogenesis	7	0,0140	3,5	4,60E-01	2,30E+01
positive regulation of CREB transcription factor activity	5	0,0140	5,1	4,60E-01	2,30E+01
ATP-dependent chromatin remodeling	6	0,0150	4	4,70E-01	2,40E+01
cellular response to vascular endothelial growth factor stimulus	6	0,0150	4	4,70E-01	2,40E+01
cell cycle	24	0,0150	1,7	4,70E-01	2,40E+01
covalent chromatin modification	15	0,0150	2	4,80E-01	2,50E+01
response to drug	31	0,0150	1,6	4,80E-01	2,50E+01
intracellular transport of virus	9	0,0170	2,7	5,00E-01	2,70E+01
heart looping	10	0,0170	2,5	5,00E-01	2,70E+01
cell migration	20	0,0170	1,8	5,00E-01	2,70E+01
interleukin-6-mediated signaling pathway	4	0,0170	6,8	5,00E-01	2,80E+01
establishment of endothelial barrier	5	0,0170	4,8	5,00E-01	2,80E+01
positive regulation of epithelial to mesenchymal transition	7	0,0180	3,2	5,20E-01	2,90E+01
beta-catenin-TCF complex assembly	8	0,0200	2,9	5,50E-01	3,10E+01
protein localization to plasma membrane	10	0,0200	2,4	5,50E-01	3,20E+01
negative regulation of signal transduction	7	0,0210	3,2	5,60E-01	3,30E+01
Golgi organization	11	0,0210	2,3	5,60E-01	3,30E+01
transmembrane receptor protein serine/threonine kinase signaling pathway	5	0,0220	4,5	5,60E-01	3,30E+01
hindbrain development	5	0,0220	4,5	5,60E-01	3,30E+01
microvillus assembly	5	0,0220	4,5	5,60E-01	3,30E+01
negative regulation of transforming growth factor beta receptor signaling pathway	10	0,0220	2,4	5,70E-01	3,40E+01
post-embryonic camera-type eye development	3	0,0230	11,5	5,80E-01	3,50E+01
protein localization to juxtaparanode region of axon	3	0,0230	11,5	5,80E-01	3,50E+01
lens fiber cell apoptotic process	3	0,0230	11,5	5,80E-01	3,50E+01
placenta blood vessel development	4	0,0230	6,1	5,80E-01	3,60E+01
organ growth	4	0,0230	6,1	5,80E-01	3,60E+01
positive regulation of cardiac muscle cell differentiation	4	0,0230	6,1	5,80E-01	3,60E+01
digestive tract development	7	0,0240	3,1	5,90E-01	3,70E+01
positive chemotaxis	7	0,0240	3,1	5,90E-01	3,70E+01
regulation of GTPase activity	10	0,0250	2,4	5,90E-01	3,70E+01
visual learning	8	0,0250	2,7	6,00E-01	3,80E+01
cell fate determination	5	0,0260	4,3	6,10E-01	3,90E+01
positive regulation of NF-kappaB transcription factor activity	16	0,0270	1,8	6,10E-01	3,90E+01
cell maturation	7	0,0270	3	6,20E-01	4,00E+01
response to cAMP	8	0,0280	2,7	6,30E-01	4,10E+01
regulation of gene expression	13	0,0290	2	6,40E-01	4,20E+01
neuron death	4	0,0310	5,6	6,50E-01	4,40E+01
positive regulation of cell proliferation	42	0,0310	1,4	6,50E-01	4,40E+01
cell-cell adhesion	27	0,0310	1,5	6,50E-01	4,40E+01
regulation of actin cytoskeleton organization	8	0,0310	2,6	6,50E-01	4,50E+01
positive regulation of sodium ion transport	5	0,0320	4	6,50E-01	4,50E+01
positive regulation of protein export from nucleus	5	0,0320	4	6,50E-01	4,50E+01
cellular sodium ion homeostasis	5	0,0320	4	6,50E-01	4,50E+01
regulation of cardiac muscle contraction by regulation of the release of sequestered calcium ion	5	0,0320	4	6,50E-01	4,50E+01
negative regulation of myeloid cell differentiation	5	0,0320	4	6,50E-01	4,50E+01
T cell receptor signaling pathway	17	0,0320	1,8	6,50E-01	4,50E+01
neuromuscular junction development	6	0,0330	3,3	6,60E-01	4,60E+01
multicellular organism growth	11	0,0350	2,1	6,80E-01	4,80E+01
positive regulation of pathway-restricted SMAD protein phosphorylation	8	0,0350	2,6	6,70E-01	4,80E+01
social behavior	8	0,0350	2,6	6,70E-01	4,80E+01
ERBB2 signaling pathway	7	0,0350	2,8	6,70E-01	4,80E+01
long-term synaptic potentiation	7	0,0350	2,8	6,70E-01	4,80E+01
protein dephosphorylation	15	0,0350	1,8	6,70E-01	4,90E+01
positive regulation of angiogenesis	14	0,0370	1,9	6,80E-01	5,00E+01
hemopoiesis	9	0,0370	2,3	6,80E-01	5,00E+01
lymphatic endothelial cell differentiation	3	0,0370	9,2	6,80E-01	5,10E+01
Type I pneumocyte differentiation	3	0,0370	9,2	6,80E-01	5,10E+01
lymphocyte differentiation	3	0,0370	9,2	6,80E-01	5,10E+01
positive regulation of endothelial cell chemotaxis	3	0,0370	9,2	6,80E-01	5,10E+01
negative regulation of pancreatic juice secretion	3	0,0370	9,2	6,80E-01	5,10E+01
behavioral fear response	6	0,0380	3,2	6,80E-01	5,10E+01
regulation of transforming growth factor beta receptor signaling pathway	5	0,0380	3,8	6,80E-01	5,10E+01
regulation of canonical Wnt signaling pathway	5	0,0380	3,8	6,80E-01	5,10E+01
transmission of nerve impulse	5	0,0380	3,8	6,80E-01	5,10E+01
regulation of cell size	5	0,0380	3,8	6,80E-01	5,10E+01
negative regulation of androgen receptor signaling pathway	4	0,0390	5,1	6,90E-01	5,20E+01
positive regulation of cell-cell adhesion	4	0,0390	5,1	6,90E-01	5,20E+01
I-kappaB phosphorylation	4	0,0390	5,1	6,90E-01	5,20E+01
lens morphogenesis in camera-type eye	4	0,0390	5,1	6,90E-01	5,20E+01
protein N-linked glycosylation	7	0,0390	2,7	6,90E-01	5,20E+01
Wnt signaling pathway, calcium modulating pathway	7	0,0390	2,7	6,90E-01	5,20E+01
transcription initiation from RNA polymerase II promoter	17	0,0390	1,7	6,90E-01	5,30E+01
positive regulation of protein catabolic process	9	0,0400	2,3	7,00E-01	5,30E+01
protein ubiquitination involved in ubiquitin-dependent protein catabolic process	17	0,0420	1,7	7,10E-01	5,40E+01
peptidyl-tyrosine phosphorylation	17	0,0420	1,7	7,10E-01	5,40E+01
erythrocyte differentiation	7	0,0440	2,7	7,20E-01	5,60E+01
pancreas development	5	0,0440	3,6	7,20E-01	5,70E+01
fat cell differentiation	10	0,0470	2,1	7,40E-01	5,90E+01
memory	9	0,0470	2,2	7,40E-01	5,90E+01
positive regulation of BMP signaling pathway	6	0,0480	3	7,50E-01	6,00E+01
regulation of endocytosis	6	0,0480	3	7,50E-01	6,00E+01
negative regulation of fibroblast proliferation	6	0,0480	3	7,50E-01	6,00E+01
cell communication by electrical coupling involved in cardiac conduction	4	0,0480	4,7	7,50E-01	6,00E+01
cardiac right ventricle morphogenesis	4	0,0480	4,7	7,50E-01	6,00E+01
dopamine receptor signaling pathway	4	0,0480	4,7	7,50E-01	6,00E+01
cardiac muscle cell proliferation	4	0,0480	4,7	7,50E-01	6,00E+01
negative regulation of neuron apoptotic process	15	0,0490	1,7	7,50E-01	6,10E+01
central nervous system development	14	0,0490	1,8	7,50E-01	6,10E+01

Table S2. Gene ontology (GO) of Biological Processes (BP) significantly associated to let-7c-5p.

Current Gene List: let-7c-5p
Current Background: Homo sapiens

Term	Count	%	P-Value	Fold Enrichment	Benjamini	FDR
protein phosphorylation	58	4,90	0,0000	2,1	1,10E-03	5,50E-04
cellular response to amino acid stimulus	14	1,20	0,0000	4,8	6,20E-03	6,50E-03
positive regulation of transcription from RNA polymerase II promoter	90	7,70	0,0002	1,5	1,70E-01	2,90E-01
negative regulation of translation	13	1,10	0,0002	3,6	1,50E-01	3,40E-01
signal transduction by protein phosphorylation	11	0,90	0,0003	4	1,80E-01	5,20E-01
transcription, DNA-templated	158	13,50	0,0003	1,3	1,60E-01	5,40E-01
collagen catabolic process	13	1,10	0,0005	3,3	2,20E-01	8,90E-01
positive regulation of activin receptor signaling pathway	5	0,40	0,0008	10,1	3,10E-01	1,50E+00
positive regulation of cell migration	24	2,00	0,0010	2,1	3,30E-01	1,80E+00
Wnt signaling pathway	24	2,00	0,0013	2,1	3,60E-01	2,30E+00
in utero embryonic development	24	2,00	0,0013	2,1	3,60E-01	2,30E+00
positive regulation of transcription, DNA-templated	50	4,30	0,0018	1,6	4,40E-01	3,20E+00
regulation of cell migration	13	1,10	0,0018	2,8	4,20E-01	3,30E+00
embryonic skeletal system development	8	0,70	0,0020	4,3	4,20E-01	3,60E+00
regulation of transcription, DNA-templated	121	10,30	0,0020	1,3	4,00E-01	3,70E+00
negative regulation of transcription from RNA polymerase II promoter	65	5,50	0,0022	1,5	4,00E-01	3,90E+00
activin receptor signaling pathway	6	0,50	0,0022	6,1	3,90E-01	4,00E+00
protein K11-linked deubiquitination	5	0,40	0,0023	8,1	3,80E-01	4,10E+00
collagen fibril organization	9	0,80	0,0023	3,7	3,60E-01	4,10E+00
palate development	13	1,10	0,0023	2,8	3,50E-01	4,20E+00
cytokinesis	10	0,90	0,0024	3,4	3,50E-01	4,30E+00
gastrulation with mouth forming second	6	0,50	0,0030	5,7	3,90E-01	5,30E+00
protein homooligomerization	22	1,90	0,0031	2	4,00E-01	5,60E+00
response to ischemia	8	0,70	0,0035	3,9	4,20E-01	6,30E+00
anterior/posterior pattern specification	13	1,10	0,0036	2,6	4,10E-01	6,40E+00
activation of JNKK activity	4	0,30	0,0041	10,8	4,40E-01	7,30E+00
positive regulation of mesenchymal cell proliferation	7	0,60	0,0044	4,3	4,50E-01	7,70E+00
positive regulation of neuron apoptotic process	9	0,80	0,0044	3,4	4,40E-01	7,70E+00
protein ubiquitination	36	3,10	0,0052	1,6	4,80E-01	9,10E+00
positive regulation of protein phosphorylation	17	1,40	0,0052	2,2	4,70E-01	9,10E+00
pre-miRNA processing	5	0,40	0,0066	6,2	5,40E-01	1,10E+01
regulation of axon extension involved in axon guidance	4	0,30	0,0068	9,2	5,40E-01	1,20E+01
negative regulation of cytoplasmic translation	4	0,30	0,0068	9,2	5,40E-01	1,20E+01
transcription from RNA polymerase II promoter	47	4,00	0,0072	1,5	5,50E-01	1,20E+01
response to X-ray	6	0,50	0,0098	4,4	6,50E-01	1,60E+01
extracellular matrix organization	22	1,90	0,0100	1,8	6,50E-01	1,70E+01
positive regulation of smooth muscle cell proliferation	10	0,90	0,0110	2,7	6,70E-01	1,80E+01
O-glycan processing	10	0,90	0,0110	2,7	6,70E-01	1,80E+01
heat generation	3	0,30	0,0110	16,1	6,60E-01	1,80E+01
targeting of mRNA for destruction involved in RNA interference	3	0,30	0,0110	16,1	6,60E-01	1,80E+01
cellular response to oxygen-glucose deprivation	3	0,30	0,0110	16,1	6,60E-01	1,80E+01
vasodilation by norepinephrine-epinephrine involved in regulation of systemic arterial blood p	3	0,30	0,0110	16,1	6,60E-01	1,80E+01
negative regulation of transcription, DNA-templated	45	3,80	0,0110	1,5	6,60E-01	1,90E+01
positive regulation of glycogen biosynthetic process	5	0,40	0,0110	5,4	6,60E-01	1,90E+01
skeletal muscle tissue development	9	0,80	0,0120	2,8	6,80E-01	2,00E+01
protein O-linked glycosylation	7	0,60	0,0130	3,5	6,70E-01	2,10E+01
activation of cysteine-type endopeptidase activity involved in apoptotic process	12	1,00	0,0130	2,3	6,80E-01	2,10E+01
positive regulation of protein kinase B signaling	12	1,00	0,0140	2,3	7,00E-01	2,30E+01
activation of MAPK activity	14	1,20	0,0150	2,1	7,10E-01	2,40E+01
regulation of sequestering of zinc ion	4	0,30	0,0150	7,2	7,00E-01	2,40E+01
branchiomotor neuron axon guidance	4	0,30	0,0150	7,2	7,00E-01	2,40E+01
response to toxic substance	12	1,00	0,0150	2,3	7,10E-01	2,50E+01
negative regulation of transforming growth factor beta receptor signaling pathway	10	0,90	0,0160	2,5	7,20E-01	2,60E+01
membrane depolarization	6	0,50	0,0170	3,9	7,20E-01	2,70E+01
inactivation of MAPK activity	6	0,50	0,0170	3,9	7,20E-01	2,70E+01
somatic stem cell population maintenance	10	0,90	0,0180	2,5	7,40E-01	2,80E+01
base-excision repair	7	0,60	0,0190	3,2	7,50E-01	3,00E+01
protein autophosphorylation	19	1,60	0,0200	1,8	7,60E-01	3,10E+01
forelimb morphogenesis	4	0,30	0,0200	6,5	7,60E-01	3,10E+01
regulation of mitochondrial membrane permeability	4	0,30	0,0200	6,5	7,60E-01	3,10E+01
regulation of gene expression	13	1,10	0,0200	2,1	7,50E-01	3,20E+01
siRNA loading onto RISC involved in RNA interference	3	0,30	0,0210	12,1	7,60E-01	3,20E+01
miRNA catabolic process	3	0,30	0,0210	12,1	7,60E-01	3,20E+01
negative regulation of aldosterone biosynthetic process	3	0,30	0,0210	12,1	7,60E-01	3,20E+01
mesenchyme development	3	0,30	0,0210	12,1	7,60E-01	3,20E+01
negative regulation of plasma membrane long-chain fatty acid transport	3	0,30	0,0210	12,1	7,60E-01	3,20E+01
negative regulation of cortisol biosynthetic process	3	0,30	0,0210	12,1	7,60E-01	3,20E+01
positive regulation of Wnt signaling pathway	7	0,60	0,0220	3,1	7,70E-01	3,30E+01
activation of MAPKK activity	8	0,70	0,0220	2,8	7,60E-01	3,30E+01
cell fate commitment	8	0,70	0,0220	2,8	7,60E-01	3,30E+01
innervation	5	0,40	0,0220	4,5	7,60E-01	3,40E+01
G1/S transition of mitotic cell cycle	13	1,10	0,0240	2,1	7,70E-01	3,50E+01
cerebellum development	7	0,60	0,0250	3,1	7,80E-01	3,70E+01
apoptotic process	48	4,10	0,0250	1,4	7,80E-01	3,70E+01
transforming growth factor beta receptor signaling pathway	12	1,00	0,0260	2,1	7,90E-01	3,90E+01
negative regulation of myeloid cell differentiation	5	0,40	0,0270	4,2	7,90E-01	3,90E+01
peptidyl-proline hydroxylation	4	0,30	0,0270	5,9	7,90E-01	3,90E+01
neuronal action potential	6	0,50	0,0270	3,5	7,80E-01	3,90E+01
negative regulation of DNA binding	6	0,50	0,0270	3,5	7,80E-01	3,90E+01
sodium ion transport	11	0,90	0,0270	2,2	7,80E-01	4,00E+01
transmembrane transport	24	2,00	0,0290	1,6	8,00E-01	4,20E+01
phosphatidylinositol-mediated signaling	13	1,10	0,0310	2	8,10E-01	4,40E+01
regulation of RNA splicing	6	0,50	0,0310	3,3	8,10E-01	4,40E+01
negative regulation of cell migration	12	1,00	0,0320	2	8,20E-01	4,50E+01
regulation of cardiac muscle cell proliferation	3	0,30	0,0340	9,7	8,30E-01	4,70E+01
radial glia guided migration of Purkinje cell	3	0,30	0,0340	9,7	8,30E-01	4,70E+01
semaphorin-plexin signaling pathway involved in axon guidance	4	0,30	0,0340	5,4	8,30E-01	4,70E+01
trophoblast giant cell differentiation	4	0,30	0,0340	5,4	8,30E-01	4,70E+01
embryonic limb morphogenesis	7	0,60	0,0350	2,8	8,30E-01	4,80E+01
positive regulation of glucose import	6	0,50	0,0350	3,2	8,30E-01	4,80E+01
post-embryonic development	10	0,90	0,0360	2,2	8,30E-01	4,90E+01
kidney development	11	0,90	0,0390	2,1	8,50E-01	5,20E+01
positive regulation of JUN kinase activity	6	0,50	0,0400	3,1	8,50E-01	5,30E+01
adult walking behavior	6	0,50	0,0400	3,1	8,50E-01	5,30E+01

intrinsic apoptotic signaling pathway in response to DNA damage by p53 class mediator	6	0,50	0,0400	3,1	8,50E-01	5,30E+01
inner ear morphogenesis	8	0,70	0,0400	2,5	8,50E-01	5,30E+01
transport	31	2,60	0,0410	1,4	8,60E-01	5,40E+01
regulation of ion transmembrane transport	13	1,10	0,0420	1,9	8,50E-01	5,40E+01
peptidyl-tyrosine dephosphorylation	12	1,00	0,0420	2	8,50E-01	5,50E+01
cardiac epithelial to mesenchymal transition	4	0,30	0,0420	5	8,50E-01	5,50E+01
pathway-restricted SMAD protein phosphorylation	4	0,30	0,0420	5	8,50E-01	5,50E+01
cell division	31	2,60	0,0430	1,4	8,60E-01	5,60E+01
positive regulation of cardiac muscle cell proliferation	5	0,40	0,0440	3,7	8,50E-01	5,60E+01
cellular response to cytokine stimulus	5	0,40	0,0440	3,7	8,50E-01	5,60E+01
brown fat cell differentiation	6	0,50	0,0450	3	8,60E-01	5,70E+01
regulation of cytokine biosynthetic process	3	0,30	0,0450	6,9	9,10E-01	7,10E+01
neural tube closure	10	0,90	0,0480	2,1	8,70E-01	5,90E+01
muscle organ development	11	0,90	0,0480	2	8,70E-01	5,90E+01
cartilage morphogenesis	3	0,30	0,0490	8,1	8,70E-01	6,00E+01
nodal signaling pathway	3	0,30	0,0490	8,1	8,70E-01	6,00E+01
transmembrane receptor protein tyrosine phosphatase signaling pathway	3	0,30	0,0490	8,1	8,70E-01	6,00E+01
collagen-activated tyrosine kinase receptor signaling pathway	3	0,30	0,0490	8,1	8,70E-01	6,00E+01
ventricular compact myocardium morphogenesis	3	0,30	0,0490	8,1	8,70E-01	6,00E+01
signaling	3	0,30	0,0490	8,1	8,70E-01	6,00E+01
positive regulation of sodium ion transmembrane transporter activity	3	0,30	0,0490	8,1	8,70E-01	6,00E+01
positive regulation of mitotic metaphase/anaphase transition	3	0,30	0,0490	8,1	8,70E-01	6,00E+01

Table S3. Molecular Mechanic-Poisson Boltzmann surface area (MM-PBSA) evaluation. MM-PBSA results for four analyzed oleocanthal (OC)-p50/p65 complexes. ΔPBSA is the sum of the electrostatic (ELE), van der Waals (VDW), polar (EPB) and non-polar (ENPOLAR) solvation free energy. Data are expressed as kcal•mol^{-1}.

	**ELE**	**VDW**	**ENPOLAR**	**EPB**	**ΔPBSA**
**Complex 1**	−31.1	−23.6	−2.6	40.6	**−16.7**
**Complex 2**	−44.1	−21.5	−2.8	48.5	**−19.9**
**Complex 3**	−36.4	−25.7	−3.2	52.2	**−13.1**
**Complex 4**	−15.6	−5.3	−2.7	31.4	**−12.2**
